# Solution structure of CXCL13 and heparan sulfate binding show that GAG binding site and cellular signalling rely on distinct domains

**DOI:** 10.1098/rsob.170133

**Published:** 2017-10-25

**Authors:** Yoan R. Monneau, Lingjie Luo, Nehru Viji Sankaranarayanan, Balaji Nagarajan, Romain R. Vivès, Françoise Baleux, Umesh R. Desai, Fernando Arenzana-Seidedos, Hugues Lortat-Jacob

**Affiliations:** 1 University of Grenoble Alpes, CNRS, CEA, IBS, 38000 Grenoble, France; 2 Institut Pasteur, INSERM U1108, Paris, France; 3 Institute for Structural Biology, Drug Discovery and Development, Virginia Commonwealth University, Richmond, VA, USA; 4 Department of Medicinal Chemistry, Virginia Commonwealth University, Richmond, VA, USA; 5 Institut Pasteur, Unité de Chimie des Biomolécules, UMR CNRS 3523, Paris, France

**Keywords:** chemokine, glycosaminoglycan, CXCL13, heparan sulfate binding site, heparan sulfate sequence

## Abstract

Chemokines promote directional cell migration through binding to G-protein-coupled receptors, and as such are involved in a large array of developmental, homeostatic and pathological processes. They also interact with heparan sulfate (HS), the functional consequences of which depend on the respective location of the receptor- and the HS-binding sites, a detail that remains elusive for most chemokines. Here, to set up a biochemical framework to investigate how HS can regulate CXCL13 activity, we solved the solution structure of CXCL13. We showed that it comprises an unusually long and disordered C-terminal domain, appended to a classical chemokine-like structure. Using three independent experimental approaches, we found that it displays a unique association mode to HS, involving two clusters located in the α-helix and the C-terminal domain. Computational approaches were used to analyse the HS sequences preferentially recognized by the protein and gain atomic-level understanding of the CXCL13 dimerization induced upon HS binding. Starting with four sets of 254 HS tetrasaccharides, we identified 25 sequences that bind to CXCL13 monomer, among which a single one bound to CXCL13 dimer with high consistency. Importantly, we found that CXCL13 can be functionally presented to its receptor in a HS-bound form, suggesting that it can promote adhesion-dependent cell migration. Consistently, we designed CXCL13 mutations that preclude interaction with HS without affecting CXCR5-dependent cell signalling, opening the possibility to unambiguously demonstrate the role of HS in the biological function of this chemokine.

## 1. Background

Chemokines are a large family of soluble chemoattractant cytokines, which control the migratory patterns and the positioning of cells through interaction with G-protein-coupled receptors [1]. Implicit in the realization of this function, they form gradients of concentration within tissues along which cells can migrate directionally. While chemokine diffusion could account for long range and oriented cell chemotactic migration, the presence of discrete sets of chemokines confined in specific tissue compartments and locally maintaining specific cell populations has suggested mechanisms restricting their diffusion and spatial distribution. Heparan sulfate (HS), present on both cell surface and within extracellular matrices [2], to which most chemokines bind, is believed to play an important role in this process [3–5].

HS is a large polysaccharide, characterized by a unique level of structural complexity. It is composed of alternating hexuronic acid, either a glucuronic acid (GlcA) or its C-5 epimer, an iduronic acid (IdoA) and an *N*-acetyl or *N*-sulfated glucosamine (GlcNAc or GlcNS). Resulting disaccharides can also be *O*-sulfated at different positions, which may occur at C-6 and occasionally C-3 of GlcN, and C-2 of IdoA or GlcA. Variations in sulfation and GlcA/IdoA ratio along the chain, generate huge microheterogeneity and diversity [6], thereby providing topologically and temporally controlled specific docking sites for the polysaccharide ligands. The functional importance of HS-mediated chemokine immobilization has remained difficult to evaluate: genetic manipulations targeting HS biosynthesis and thus structure have broad effects because they also affect the interaction of HS with many other proteins; additionally, possible overlaps of the chemokine domains involved in receptor-mediated cell signalling and HS binding may obscure the interpretation of the results obtained using HS binding mutant chemokines, which would display reduced agonist capacity.

Nevertheless, previous work using CXCL12, which features functionally and spatially non-overlapping HS and receptor binding site [7] as a model system, has unambiguously demonstrated the role of HS-mediated immobilization in orchestrating the proper migration of many cell types [8,9]. Transgenic mice expressing a *Cxcl12* variant, in which mutations that preclude interaction with HS while not affecting CXCR4-dependent cell signalling, showed an increased level of free CXCL12 correlated with dysfunction in angiogenesis and tissue neovascularization [9]. This model also demonstrated that HS-immobilized CXCL12 was essential for the correct positioning of B-lymphocytes within germinal centres and the production of high-affinity antibodies [8].

CXCL13 also importantly regulates the migration and positioning of T- and B-lymphocytes in secondary lymphoid organs, where it cooperates critically with CXCL12 in the formation of germinal centres [10]. Although CXCL13 binding to HS has not been yet studied, it is tempting to hypothesize that, as CXCL12, it can be functionally segregated and maintained in specific area through another HS subset mediated immobilization. Here, to provide a framework in which to investigate how HS can regulate CXCL13 activity and enable the design of an *in vivo* model to analyse the functional consequences of this interaction, we have solved the structure of this chemokine and fully characterized its HS binding determinants. We report that CXCL13 features a chemokine-like fold to which is appended a non-structured 19-amino-acid-long C-terminal extension. Two domains, located within the C-terminal α-helix and within the unusual C-terminal extension of the chemokine cooperatively contribute to the interaction. Using computational approaches, we identified HS tetrasaccharide sequences that preferentially interact with CXCL13 and deduced a model describing how such sequences promote CXCL13 dimerization. Importantly, we observed that mutant-CXCL13 that does not bind to HS remained fully active. Consistently, unliganded or HS-bound CXCL13 have similar signalling, demonstrating that it can be functionally presented in an HS-bound form to its receptor. The observation that the HS binding site can be manipulated without affecting the receptor-dependent biological activity of this chemokine opens the route towards the analysis of the contribution of HS binding in the regulation of the CXCL13 bioactivity. Additionally, from a structural point of view, we observed that the presence of the CXCL13 initiating N-terminal methionine, resulting from its expression in *Escherichia coli*, unexpectedly strongly affect the folding of the N-terminal region of the protein. As this region is critically involved in the signalling activity of all chemokines, careful attention should be paid to the expression system used for chemokine preparation.

## 2. Material and methods

### 2.1. Wild-type and mutant-CXCL13 expression and purification

Murine CXCL13 (residues 22–109) and ΔN-CXCL13 (residues 31–109) were cloned into a pET-17b expression vector, resulting in protein constructs of 89 and 80 amino acids, respectively, including the initiating methionine. A CXCL13 construct, including an enterokinase cleavage site was also designed to produce CXCL13 without the first methionine (see the electronic supplementary material). CXCL13-ΔC (residues 22–95) lacking the last 14 amino acids was produced by introducing a stop codon at the corresponding position. Mutations were incorporated by one-step site-directed mutagenesis. Plasmids were characterized by DNA sequencing and used to transform *E. coli* BL21-codon Plus strain. Cells were grown at 37°C and induced with 0.5 mM isopropyl 1-thio-β-d-galactopyranoside for 3 h, after which CXCL13, expressed as inclusion body, was refolded and purified using anion exchange and gel filtration chromatography as detailed in the electronic supplementary material.

### 2.2. NMR spectroscopy

NMR experiments were performed as detailed in the electronic supplementary material. Briefly, ^1^H, ^13^C and ^15^N chemical shift assignment of CXCL13 was performed on 200 µM protein samples in 20 mM phosphate/100 mM NaCl pH 6 buffer at 298 K using standard triple resonance experiments. The residues involved in CXCL13 dimerization or binding to HS were determined by calculating the combined ^15^N and ^1^H CSPs as previously described [11]. Dimerization equilibrium constant (*K*_D_) was calculated from specific integrated NMR signals of both monomer and dimer. The longitudinal (T1) and transverse (T2) ^15^N relaxation times and the heteronuclear (^1^H)-^15^N steady-state NOE were measured for Met-CXCL13 at 680 µM and at 298 K on ΔN-CXCL13 at 750 µM, using previously published methods and pulse sequences [12]. The tumbling correlation time *τ*_c_ was calculated based on the T1/T2 ratio according to the equation described by Kay *et al*. [13] and molecular weights (MWs) for CXCL13 at different oligomeric states were estimated as described [14].

The structure calculation was achieved using 1683 unassigned NOE-based distance restraints extracted from 3D ^15^N- and ^13^C-edited-NOESY experiments with mixing times set to 120 ms, along with two leucine (Leu62 and Leu66) residue χ_2_ dihedral angles, determined according to the difference between C_δ1_ and C_δ2_ resonances [15], 90 backbone dihedral angles obtained from TALOS-N and 14 hydrogen bonds when consistent with NOE patterns and only for amides displaying slow hydrogen–deuterium exchange. The structure ensemble was calculated using a standard CYANA 3.97 set-up [16] prior to energy minimization in the presence of water for the 20 best-issued structures achieved with CNS water-refinement scripts along with 843 distances (based on NOE, hydrogen bonds and disulfide bridges) and 90 dihedral angles [17] and has been deposited in the Protein Data Bank with the accession code 5L7M.

### 2.3. Molecular model of CXCL13 dimer

The homodimer CXCL13 was modelled using HADDOCK (High Ambiguity-driven protein–protein docking) webserver (http://haddock.science.uu.nl/services/ HADDOCK2.2) in which the solution structure of monomeric CXCL13 along with CSP data of dimer were used. The ambiguous interaction restraints (AIRs), which consist of active and passive residues, were defined from the CSP data of the dimer. The resulting model was further confirmed by inter-molecular NOEs.

### 2.4. HS binding sites identification

GAG binding site sequencing was performed as previously described [18]. Briefly, heparin beads were activated with 40 mM l-ethyl-3-(3-dimethylaminopropyl) carbodiimide and 10 mM *N*-hydroxysuccinimide, in 50 mM MES, 150 mM NaCl pH 5.5, for 10 min at room temperature and incubated with 25 µg of recombinant Met-CXCL13 in PBS for 2 h at room temperature (final concentration of 3 µM). The reaction was stopped by addition of 1 M Tris, pH 7.5 (100 mM Tris, final concentration) and the covalently bound-CXCL13 was denatured with 2 M urea and 75 mM ß-mercaptoethanol, at 60°C for 45 min, prior to overnight digestion with thermolysin (50 mIU) in PBS at 50°C. Released peptides were removed by washing the beads with PBS modified with 2 M NaCl, 75 mM β-mercaptoethanol and 1% Triton, while cross-linked peptides, overlapping the CXCL13 GAG binding sites, were identified by Edman degradation automated sequencing performed directly on the beads.

Chemical shift mapping was also achieved to identify the CXCL13 binding surface involved in HS recognition. To proceed, sample containing 100 µM of ^15^N-labelled Met-CXCL13 in 20 mM potassium phosphate and 100 mM NaCl at pH 6 was mixed with HS-derived dp4 (prepared as described in the electronic supplementary material) at a final concentration of 37, 74 and 144 µM. One ^15^N-SOFAST-HMQC [19] was recorded on each sample and the combined chemical shift perturbation of *i*th residue was calculated as described in NMR spectroscopy paragraph. Use of Met-CXCL13, that does not dimerize as easily as CXCL13 upon binding to HS, considerably simplified the spectra and enabled to identify without ambiguity the residues specifically perturbed by the binding to HS.

### 2.5. Surface plasmon resonance based binding assay

Two flow cells of a CM4 sensorchip were functionalized with 2000–2500 resonance units (RU) of streptavidin as described [20]. Biotinylated HS (prepared as described in the electronic supplementary material) was captured to a level of 50 RU on one surface; the other one was left untreated and served as negative control. Before use, the chip was washed by continuous flow of HBS-P buffer (Hepes 10 mM, NaCl 0.15 M, P20 detergent 0.05%, pH 7.4). For binding assays, a range of concentrations of wild-type or mutant-CXCL13 was injected both over negative control and HS surfaces for 5 min at 25°C and 50 µl min^−1^, with the following conditions: Met- and ΔN- CXCL13: 12, 18, 28, 41, 62, 93 and 140 nM; C5-mutant: 15, 22, 33, 50, 74, 112 and 167 nM; C1-mutant: 50, 74, 112, 167, 251, 377 and 565 nM; and C1.5-mutant: 86, 129, 193, 289, 434, 651 and 977 nM. The HS surface was regenerated with a 250 µl pulse of 2 M NaCl. Control sensorgrams were subtracted online from HS sensorgrams. The association and dissociation rate constants (*k*_on_ and *k*_off_) were determined by fitting both the association and dissociation phases for each CXCL13 concentration to a 1 : 1 binding model using the biaeval v. 3.1 software, from which the equilibrium dissociation constant (*K*_D_ = *k*_off_/*k*_on_) was calculated.

### 2.6. Heparan sulfate tetrasaccharide structure preparation and CXCL13-HS molecular modelling and dynamic studies

Disaccharides consisting of both iduronic acid (IdoA, ^1^C_4_ and ^2^S_O_ conformations) and glucosamine (GlcN, ^4^C_1_ conformation) were used to construct the library of common tetrasaccharide (dp4) sequences. The coordinates for these sequences were generated in a combinatorial manner using a previously developed protocol called combinatorial virtual library screening (CVLS) strategy [21,22]. The library consisted of two sets of 254 dp4 sequences made combinatorially starting with either IdoA or a GlcN at the non-reducing end.

The coordinates of the solution structure of CXCL13 were prepared for molecular modelling using SYBYL-X v. 2.1. This involved addition of hydrogen atoms, alteration of protonation states of amino acids to physiological conditions and energy minimization of the structure. Based on our experimental observations using NMR, we defined two potential HS binding sites, binding site I (cluster 1), which included α-helix, and binding site II (cluster 5), which included C-terminal extension region. Key residues Arg58, Lys60, Arg64, Arg67 and Lys72 were enclosed with cluster 1 and Lys84, Arg85 and Arg86 were part of cluster 5. Both sites enveloped a radius of 16 Å and could easily accommodate tetrasaccharide sequences.

Molecular docking of each tetrasaccharide sequence at the two putative binding sites was performed with GOLD v. 5.2 (Cambridge Crystallographic Data Centre, Cambridge, UK) using our dual filter algorithm strategy [22]. Best sequences were identified as those with the highest GOLDScore (*in silico* ‘affinity’) and the lowest root-mean-square deviation (*in silico* ‘specificity’) between the top six solutions derived from a triplicate docking run for each sequence and the hydrogen-bond formation with key residues.

The selected CXCL13–dp4 complexes were used as initial structures for unrestrained constant temperature and pressure molecular dynamic simulation as detailed in the electronic supplementary material. Hydrogen bonding, water mediating interaction between dp4 and CXCL13 residues, binding free energy calculation of each co-complex and single-residue energy decomposition (SERD) to estimate the energy contribution of each single-residue responsible to the bound state were computed and analysed.

### 2.7. Chemotaxis and intracellular calcium release assays

Chemotaxis assays were performed on freshly isolated murine splenocytes, extracted from two-month-old BALB/c mice. Murine spleens, soaking in RPMI 1640 buffer supplemented with 20 mM Hepes and 1% Bovine Serum Albumin (BSA), were mashed out using a 40 µm cell strainer. Splenocyte suspension was passed through a 100 µm cell strainer prior to incubation for 30 min at 37°C and 5% CO_2_. Cell migration was assessed in triplicate in 98-well chamber Transwell plates with 5 µm pores. Splenocyte suspension (80 µl, 1.5 × 10^5^ cells) was dropped into each insert while 235 µl of chemokine in RPMI 1640 buffer supplemented with 20 mM Hepes and 1% BSA were poured into each lower well. Splenocytes that migrated into lower wells after 3 h of incubation were counted using flow cytometry (FACScantoTMII Flow Cytometer, BD Bioscience, USA).

For calcium release assays, 4 × 10^5^ CHO-pgs 677 cells were first transduced with 600 ng p24 of lentiviral vector particles of murine CXCR5 in complete HAM F12 medium. Then 3 × 10^4^ cells, which CXCR5 expression was monitored by FACS scan analysis (electronic supplementary material, figure S10), were incubated with 4 µM Fura-2 AM, diluted with HBSS Ca^2+^ Mg^2+^ in the dark at 37°C 5% CO_2_ for 1 h, plated in each channel of a Ibidi μ-Slide VI 0.4 system, washed with HBSS Ca^2+^ Mg^2+^ and activated with CXCL13 at 37°C. Imaging was performed with an Eclipse TE300 (Nikon) inverted microscope, equipped with a X10 UV-permissive objective, in a 37°C regulated chamber. Fura 2-AM-loaded cells were excited at 350 and 380 nm. Emission at 510 nm was used for analysis of the Ca^2+^ responses and images were obtained with a cooled charge-coupled device camera (CoolSNAPfx; Roper Scientific) and the Metafluor acquisition imaging software (Universal Imaging). Statistical analysis was conducted using the prism v. 5.0 software.

## 3. Results

### 3.1. Solution structure of the monomeric CXCL13 reveals a canonical chemokine shape to which is appended a fully flexible C-terminal tail

**Figure 1.**
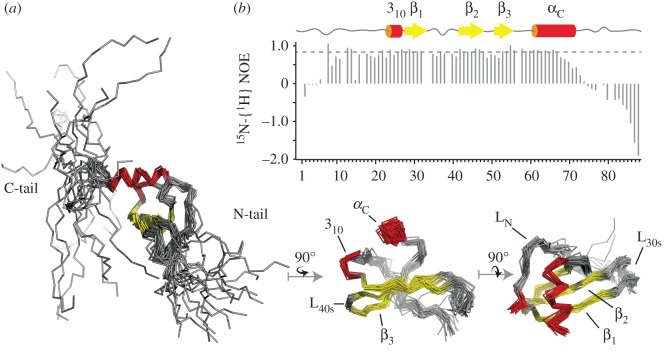
Solution structure of CXCL13. (*a*) The solution structure ensemble of wt-CXCL13 is displayed as ribbon and structure elements are notified, including the three β-strands (in yellow), the two helices (α_C_ and 3_10_ in red), the loops (L_N_, L_30S_ and L_40S_) and the two N- and C-terminal tails (N- and C-tail). From left to right, the structures are displayed along an axis parallel to β-strands, parallel to α-helix axis or normal to the β-sheet plane. For clarity, the two flexible N- and C-terminal sequences are only display on the left view. (*b*) ^15^N-{^1^H} NOEs were measured at 291 K on Met-CXCL13. The dashed line highlights the average values for the core protein residues.

**Figure 2.**
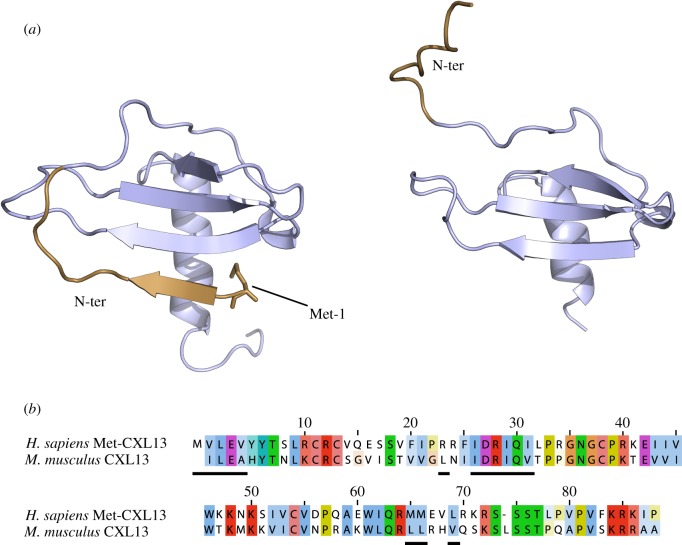
Comparison of human Met-CXCL13 and murine wt-CXCL13 sequences and structures. (*a*) Structure of human Met-CXCL13 (left, PDB ID 4ZAI) and murine wt-CXCL13, in which the initiating Met residue was removed (right, PDB ID 5L7M), depicted as cartoon, illustrating the differences in N-terminal domain (in brown) folding. (*b*) Sequence alignment of the human and murine CXCL13, used for structure resolution, depicted with a colour and intensity code according to residue type and homology (prepared with ClustalW). The residues involved in the N-terminal domain folding (1–4, 23, 26–31, 65–66 and 69) are underlined.

Recombinant ^15^N- and ^13^C-labelled murine CXCL13 was purified and processed so as to eliminate the additional N-terminal Met residue. Its backbone resonances were assigned using standard triple resonance-type experiments (see electronic supplementary material, figure S1A for the assigned HSQC) and its solution structure solved using torsion angle dynamics preceding rotamer refinement in the presence of water, along with NOE-based distance, hydrogen bond and backbone dihedral angle constraints. The summary of conformationally restricting constraints and structure quality factors are reported in table 1 for the CXCL13 monomer structure ensemble, which was deposited in the Protein Data Bank (pdb ID 5L7M). CXCL13 displays the overall canonical shape of CXC-chemokine type consisting of a disordered N-terminal sequence that, in agreement with ^15^N-{^1^H} NOE values, goes up to Lys10, a long N-terminal loop, a 3_10_ helix, and a triple-stranded anti-parallel β-sheet overlaid by a C-terminal α-helix. Additionally, CXCL13 also includes, downstream of the α-helix, a 19 residue C-terminal extension that remains unfolded and conformationally dynamic (figure 1). Accordingly, this C-terminal extension does not interact with the core domain of the protein, as confirmed with the analysis of a C-terminally truncated form of CXCL13 lacking the last 14 amino acids, (CXCL13-ΔC), which showed no disturbance of the core protein NMR signals compared with that of the full-length protein (electronic supplementary material, figure S2). This result contrasts with an X-ray crystal structure of the human CXCL13, recently deposited in the pdb (ID 4ZAI), for which the N-terminal residues adopt a β-strand conformation in a parallel association with the core β-sheet of the chemokine (figure 2*a*). Considering that the human and murine CXCL13 share a high sequence homology (70%) for both the residues of the N-terminal domain and of the β-sheet involved in this particular N-terminal structure (figure 2*b*), a similar folding would have been expected. It is worth noting, however, that the human CXCL13 construct includes the extra initiating methionine, in position −1. In the crystal, this methionine interacts with the core domain of the protein and thus could contribute to the unusual N-terminal structure stabilization. To re-assess this unusual observation, we solved the structure of the murine CXCL13 containing an extra initiating methionine and characterized the dynamics and the structure of the N-terminal domain. We found that this murine construct features a β-strand-structured N-terminal tail in a parallel association with the core β-sheet of the chemokine, in slow exchange with a flexible state (electronic supplementary material, figure S3). Thus, in the case of the murine CXCL13, the presence of a methionine at the −1 position is enough to trigger a completely different and presumably artefactual behaviour of the N-terminal residues, which was not detected in the wt-murine CXCL13 (electronic supplementary material, figure S1B).

**Table 1.** Summary of conformationally restricting constraints and structure quality factors for the CXCL13 ensemble.^a^

*completeness of resonance assignments of CXCL13*[22–109]^b^			97.1%
*conformationally restricting restraints*^c^			
	NOE restraints	total	797
		intra-residue (*i* = *j*)	252
		sequential (|*i* − *j*| = 1)	248
		medium range (1 < |*i* − *j*| < 5)	124
		long range (|*i* − *j*| ≥ 5)	171
		NOE restraints/restrained residue	9.4
	hydrogen-bond restraints		28
	dihedral angle restraints		94
	total number of restraints		919
	number of restraints/restrained residue (total/long range)		10.9/2.2
*residual restraint violations*^c^			
	average distance restraint violations/structure	0.1–0.2 Å	19.2
		0.2–0.5 Å	2.5
		>0.5 Å	0.0
	average RMS of distance violation/restraint (Å)		0.03
	maximum distance violation (Å)		0.29
	dihedral angle violations/structure	1–10°	11.05
		>10°	0
	RMS of dihedral angle violation/constraint (°)		1
	maximum dihedral angle violation (°)^d^		9.4
*model quality*^c^			
	RMSD from average coordinates (Å)	all backbone atoms (ordered/all)	0.9/8.3
		all heavy atoms (ordered/all)	1.5/8.9
	RMSD bond lengths (Å) for all residues		0.02
	RMSD bond angles (°) for all residues		1.2
	molprobity Ramachandran plot^d^	most favoured regions (%)	99.2
		additionally allowed regions (%)	0.2
		disallowed regions (%)	0.6
	global quality scores (raw/*Z*-score)^d^	Verify3D	0.23/−3.69
		ProsaII	0.31/−1.41
		Procheck G-factor (*ϕ*,*ψ*)^d^	−0.34/−1.02
		Procheck G-factor (all dihedrals)^d^	−0.29/−1.71
		MolProbity clashscore	4.83/0.70

^a^Structural statistics were computed for the ensemble of 20 deposited structures (PDB ID 5L7M).

^b^Computed for residues of CXCL13[22–109]. Observable resonances consistent to *U*-[^15^N,^13^C].

^c^Calculated for protein using PSVS 1.5 program (http://psvs-1_5-dev.nesg.org). Average distance constraints were calculated using the sum of *r*^−6^.

^d^Ordered residue ranges [*S*(*ϕ*) + *S*(*ψ*) > 1.8] : 11–13, 16–33, 37–69. Secondary structure elements: 26–32 (β1), 42–47 (β2), 52–55 (β3) and 60–68 (αC).

### 3.2. CXCL13 interacts with heparan sulfate through both its α-helix and C-terminal domain

**Figure 3.**
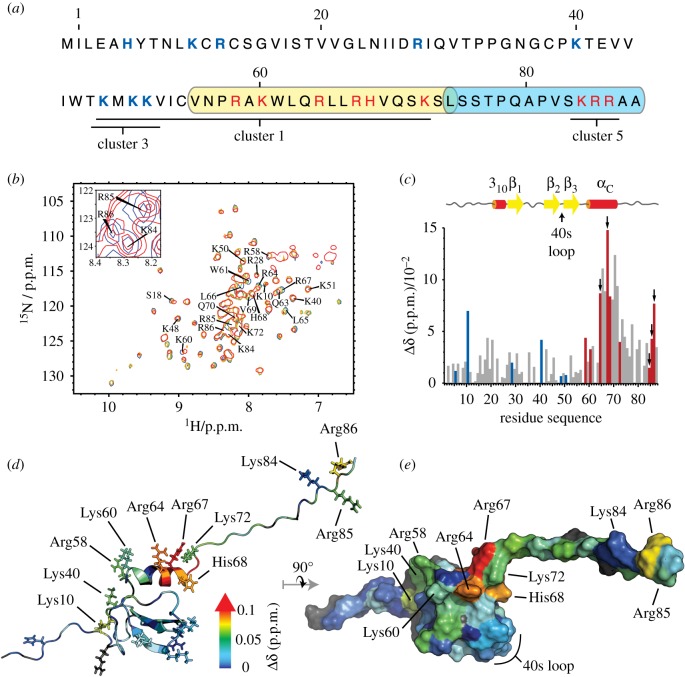
CXCL13-HS binding sites. (*a*) CXCL13 sequence highlighting the three putative HS binding clusters 1, 3 and 5 (underlined). The coverage of CXCL13 sequence by the two heparin-covalently bound peptides (yellow and blue boxes) includes nine basic residues (in red) and matches the above-mentioned predicted clusters 1 and 5 binding sites. (*b*) The [^15^N,^1^H]-correlation spectra recorded on Met-CXCL13 at 37 µM alone (blue) or in presence of 37 µM (green), 74 µM (orange) or 144 µM (red) of dp4 have been overlaid to highlight the CSPs of CXCL13 amides upon binding to HS. The inset displays the spectra recorded on sample without (blue) or with 144 µM of dp4 (red) to highlight the CSPs experienced by the C-terminal basic cluster. (*c*) The combined CSPs of CXCL13 (37 µM) amides upon binding to HS dp4 (74 µM) were plotted against the amino acid sequence number. Coloured CSPs correspond to the blue and red residues in (*a*). Basic residues experiencing large CSPs, which are thus likely to directly interact with HS (arrows), were selected as candidates for point mutations. (*d,e*) Cartoon or surface representation of CXCL13 structure, on which CSP values are mapped using a blue to red colour gradient from 0 to a specified threshold, above which the colour was set red. The most affected residues are localized within the α-helix and C-terminal flexible tail. In opposition, the 40s-loop-included residues (highlighted in *e*) remained unaffected upon dp4 binding.

It is well documented that binding of all chemokines to HS is highly relevant to their function [3]. Electrostatic forces usually contribute significantly to such interactions, and binding sites are thus commonly enriched in basic residues. Within the chemokine family, HS binding sites have been localized to five distinct regions, called clusters 1–5. Examination of the murine CXCL13 primary sequence (figure 3*a*) reveals the presence of three groups of basic amino acids, located in the 40s loop (shared by cluster 3 type HS binding sites) found in most CCL chemokines, in the C-terminal α-helix (cluster 1 type HS binding sites) found in most CXCL chemokines [23] and in the C-terminal sequence that extends downstream of the α-helix (cluster 5 type). Up to now, the cluster 5 HS binding site has been identified in CCL21, CXCL9 and CXCL12γ only [3]. To analyse the possible involvement of these clusters in HS recognition, we first used a specific experimental technique based on the formation of cross-linked heparin-protein complexes, the proteolytic digestion of these complexes and the identification of the polysaccharide-covalently bound peptides by N-terminal sequencing. Cross-linked residues can be easily identified, as they are not eluted by Edman sequential degradation and therefore yield a ‘blank’ cycle during the sequencing [18]. Analysis of CXCL13 using this approach and incubated at 3 µM typically yielded two peptides, corresponding to the α-helix and the C-terminal domain of CXCL13: V_55_NPRAKWLQRLLRHVQSKSL_74_ and L_74_SSTPQAPVSKRRAA_88_, with cross-linking at the level of Lys60 for the former, and Lys84 or Arg85 or Arg86 for the latter (figure 3*a*).

To get further insights into the HS binding surface, we next used NMR spectroscopy to follow the chemical shift perturbations (CSP) of CXCL13 backbone amides (figure 3*b*) upon titration with HS-derived tetrasaccharides (dp4). In agreement with the above described results, the presence of the dp4 affected two areas (figure 3*c*–*e*), one mainly including the basic residues Arg64 and Arg67 and their surrounding residues (Arg58, Lys60) within the α-helix (cluster 1 type) and the other, the basic residues Arg85 and 86 of the C-terminal extension (cluster 5 type). A few additional residues, such as K10 and K40, were also affected (albeit to a smaller extent). However, although CXCL13 was incubated at high concentration (3 µM) in the cross-linking approach, a method that was shown to identify low affinity binding sites [18], these residues were never picked up, ruling out they could play a significant role in HS recognition. Finally, non-basic residues also experienced CSPs, such as Leu65 and Leu66, for example (figure 3*e*). These residues, which are pointing towards the protein core, presumably underwent conformational reorganization rather than direct binding with the ligand. Surprisingly, but consistent with the sequencing approach, the 40s loop K_48_MKK_51_ motif, although solvent accessible and typically corresponding to a cluster 3 HS binding motif, was not affected by the dp4 (see ‘Discussion and conclusion’ section).

### 3.3. Clusters 1 and 5 differentially contribute to the stability and the association rate of the CXCL13-HS complex

**Figure 4.**
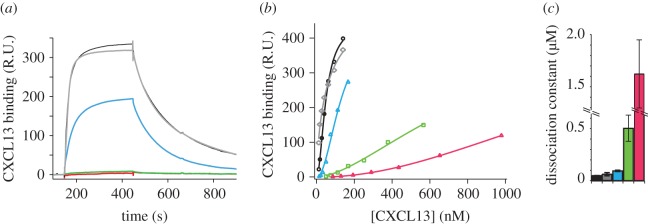
SPR analysis of the CXCL13-HS interaction. (*a*) Met-CXCL13 (black), ΔN-CXCL13 (grey) and C5- (blue), C1- (green) and C1.5- (red) mutant Met-CXCL13 (100 nM) were flowed over a HS functionalized surface and the binding response (in RU) was recorded as a function of time (in seconds). (*b*) Met-CXCL13 and ΔN-CXCL13 (12–140 nM), C5-CXCL13 (15–167 nM), C1-CXCL13 (50–565 nM) and C1.5-CXCL13 (86–980 nM) were injected over the HS surface and the binding responses were reported against CXCL13 concentration. (*c*) The binding responses were fitted as described in the material and method section to calculate the dissociation constant. The colour coding in (*b*) and (*c*) is as in (*a*).

To confirm the involvement in HS binding of clusters 1 and 5 and analyse their respective contributions, we next designed three mutants containing the [R_65_LLR_68_] to [ALLA] mutations (C1-CXCL13), the [K_85_R_86_R_87_] to [AAA] mutations (C5-CXCL13), or mutations in both sites (C1.5-CXCL13). We first verified that none of the mutations of clusters 1 and 5 either individually or in combination have impact on the protein structure (electronic supplementary material, figure S4). To validate the altered ability of the CXCL13 mutants to bind HS, we monitored their interaction using a solid-phase assay that mimics the cell membrane-anchored HS. For that purpose, reducing end biotinylated HS was immobilized on top of a streptavidin-coated sensorchip, and surface plasmon resonance (SPR) monitoring was used to measure binding when CXCL13 samples were flowed across the HS surface. Binding curves, obtained when 100 nM of wt- or mutant-CXCL13 were injected over the HS surface, first demonstrated that CXCL13 is an HS binding protein. Mutations within the α-helix (C1-CXCL13) abrogated binding while that in the C-terminus (C5-CXCL13) reduced the interaction, indicating that the α-helix is the main binding determinant (figure 4*a*). The binding curve obtained when ΔN-CXCL13 was injected over the HS surface was very similar to that of Met-CXCL13, showing that the structure of the N-terminal domain has no influence on this interaction. Visual inspection of the sensorgrams also suggested that the Met- and the Met-C5-CXCL13 dissociate from HS with similar off-rates. To further assess the contribution of the different HS binding domains, a range of concentrations of Met-, ΔN- and C5-CXCL13 were flowed across the HS surface and the resulting binding curves were fitted to a Langmuir binding model (electronic supplementary material, figure S5). These analyses returned dissociation rate constants (*k*_off_) of 5.8 ± 0.34 × 10^−3^, 5.3 ± 0.24 × 10^−3^ and 7.5 ± 0.04 × 10^−3^ s^−1^ for the Met-, ΔN- and Met-C5-CXCL13 indicating indeed that the HS binding site, localized within the C-terminal extension, minimally contributes to the stability of the complex. By contrast, mutations within this domain decreased the average association rate constants (*k*_on_) from 3.08 ± 0.05 × 10^5^ M^−1^ s^−1^ for the Met-CXCL13 (or 2.7 ± 0.6 × 10^5^ M^−1^ s^−1^ for ΔN-CXCL13) to 9.14 ± 0.58 × 10^4^ M^−1^ s^−1^ for C5-CXCL13. The Met- ΔN- and Met-C5- CXCL13 thus display *K*_D_ (=*k*_off_/*k*_on_) of 19, 19.6 and 82 nM, respectively, for HS. To determine affinity independently of the kinetic aspect of the interaction, binding isotherm was built from the steady-state data (electronic supplementary material). Binding affinities of 50 ± 3 nM and 95 ± 9 nM were calculated for wt- and C5-CXCL13, respectively, in relatively good agreement with the kinetic analysis. The affinity of the C1-mutant (mutation within the α-helix) was 504 ± 126 nM, while that of the double mutant (C1.5-CXCL13) was reduced to 1.62 ± 0.33 µM (figure 4*b*,*c*). Altogether, these analyses confirm that while both sites cooperatively contribute to the interaction, the cluster 1 (α-helix) features the main binding determinants and that the N-terminal domain structure (which is involved in bioactivity—see below) has no effect of HS recognition.

### 3.4. Analysis of CXCL13 binding to HS tetrasaccharides

We next used computational techniques to gain atomic-level understanding of HS binding to CXCL13. To investigate the interaction of HS sequences to CXCL13, we used a docking protocol called CVLS algorithm, which has been previously established for sulfated GAGs [22]. This approach was applied to four sets of 254 sequences including RE-IdoA-GlcN-NRE and RE-GlcN-IdoA-NRE to both clusters 1 and 5 (RE, reducing end; NRE, non-reducing end). Docking of each sequence to both clusters was carried out using 100 genetic algorithm (GA) runs and 100 000 iterations and the highest-ranking 25 sequences (approx. top 10%) by GOLDSCORE from each library were re-docked in triplicate for evaluating the consistency of binding. The RMSD between the six best poses of each sequence was calculated and those sequences with RMSD values of less than 2.5 Å were designated as ‘specific’ binding partners. This analysis led to identification of five sequences of RE-IdoA-GlcN-NRE type and nine sequences of RE-GlcN-IdoA-NRE type for cluster 1 as ‘specific’ dp4 structures. Likewise, four sequences of RE-IdoA-GlcN-NRE and 17 sequences of RE-GlcN-IdoA-NRE type were discovered as ‘specific’ dp4 structures for cluster 5.

**Figure 5.**
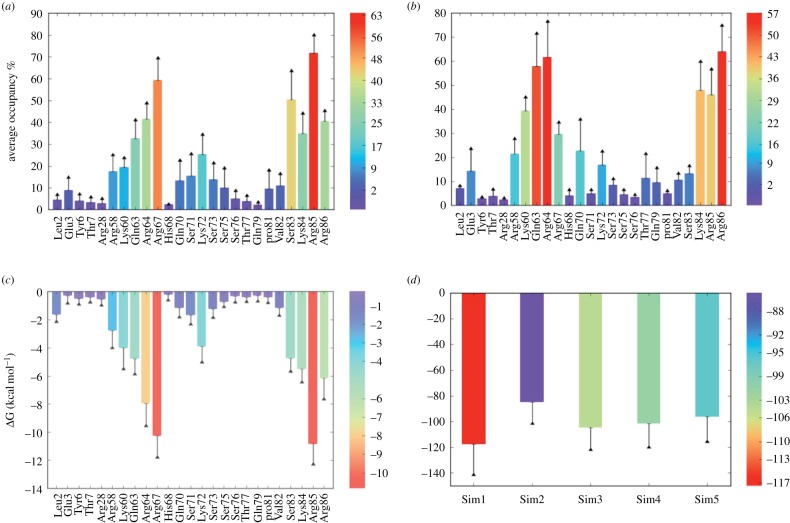
CXCL13–dp4 binding studies from MD simulations. (*a*) Average inter-molecular hydrogen-bond occupancy for dp4 binding residues in clusters 1 and 5, from all five performed simulations. (*b*) Average occupancy of water-mediated interaction between CXCL13 and dp4 residues in the interacting regions of clusters 1 and 5 from all the five simulations. (*c*) Single-residue energy decomposition values for CXCL13–dp4 interaction from clusters 1 and 5 regions. (*d*) Total binding free energy values with standard deviation as error for the CXCL13–dp4 complex from all the five simulations. For better understanding, the values from lower to higher are represented by rainbow colour bars from blue to red, respectively. The error bar shows the standard error of the mean.

To further parse most favoured sequences (table 2), we used a hydrogen-bond filter, which evaluated formation of a hydrogen bond with residues of the two clusters. Sequences with a higher level of hydrogen bonding were taken up for 50 ns MD simulation studies, performed in the presence of explicit TIP3P water molecules, where both clusters 1 and 5 were simultaneously occupied by a dp4 sequence. We averaged the overall conformational behaviour of dp4 sequences by using the results from five independent stable simulations (total time = 250 ns). In many MD runs, significant conformational plasticity was observed for dp4 sequences in which inter-molecular hydrogen-bond exchanges were found between neighbouring sugar residues (IdoA to GlcN) with a corresponding receptor residue (e.g. Arg64, Arg67 in cluster 1 and Arg84, Arg85 in cluster 5). At the same time, a specific stable inter-molecular hydrogen bond between a distinct saccharide (either IdoA or GlcN) and a distinct residue (such as Arg64, Arg67 and/or Arg86) was also observed for a few sequence combinations (results not shown). To quantify these interactions, the inter-molecular hydrogen-bond formation was analysed using the VMD hydrogen-bond plugin [24]. Figure 5*a* shows the average occupancy of inter-molecular hydrogen bonds from all the five simulations. Arg64 and Arg67 from cluster 1 and Arg85 from cluster 5 showed occupancy of more than 50% indicating the presence of at least one hydrogen bond throughout the simulation. While other vicinal residues including Arg58, Lys60 and Gln63 from cluster 1 and Lys84 and Arg86 from cluster 5 displayed occupancies in the range of 20–50% implying formation of a transient hydrogen bond. The overall inter-molecular hydrogen-bond occupancy from cluster 1 residues was approximately 30% more than that for cluster 5 residues indicating that, consistent with the experimental data, cluster 1 residues contribute more towards stability of the complex. The conformational fluctuations of the CXCL13 N- and C-terminal regions were extensive during the simulation and three sample orientations that are significantly distinct are shown in electronic supplementary material, figure S6A–C.

**Table 2.** HS tetrasaccharide sequences obtained from CVLS, bound to CXCL13 for binding sites I and II.

binding site	tetrasaccharide sequence	RMSD (Å)	GOLD score	no. H-bond	interacting residues
*α-helix* (cluster 1)	IdoA-GlcNAc6S-IdoA-GlcNS6S	1.23	76.80	7	R58, R64, K72
	IdoA2S-GlcNS6S-IdoA2S-GlcNS	1.52	74.48	12	K60, R64, R67, K72
	GlcNS6S-IdoA-GlcNAc6S-IdoA2S	1.15	102.49	14	R58, Q63, R64, R67, Q70, K72
	GlcNAc6S-IdoA2S-GlcNS6S-IdoA2S	1.61	78.84	10	K60, Q63, R64, R67, Q70, K72
*C-ter* (cluster 5)	IdoA-GlcNS-IdoA2S-GlcNS6S	1.70	53.42	4	K84, R85, R86
	IdoA2S-GlcNS6S-IdoA2S-GlcNS6S	1.30	41.50	7	S83, R85, R86, A87, A88
	GlcNS6S-IdoA2S-GlcNAc6S-IdoA2S	1.63	54.35	10	S83, K84, R85, R86, A87
	GlcNS6S-IdoA-GlcNS6S-IdoA2S	1.19	49.62	12	S83, K84, R85, R86, A87

HS are also highly water bound and typically possess multiple water-mediated interactions with proteins [25]. Such intervening water molecules can play additional key roles in stabilizing the co-complex. Electronic supplementary material, figure S6D shows the bed of water molecules forming a complex intervening network [26], whereas figure 5*b* displays the average occupancy of these water-mediated hydrogen bonds. Arg64, Arg67 and Arg86 showed maximum water-mediated hydrogen bonding. Cluster-1-included residues were 75% more likely to hold intermediate water molecules than cluster-5-included residues further explaining their main stability-governing role.

To understand residue specific interactions, we performed SERD to identify top anchoring residues for both clusters. Arg67 and Arg85 contributed the maximum energy of −10 kcal mol^−1^ towards the binding (figure 5*c*). The overall sum of SERD for binding partners from both the regions indicated that cluster 1 residues energetically contribute nearly 8 kcal mol^−1^ higher energy than that of cluster 5 residues, in agreement with the experimental data. Figure 5*d* shows the total binding free energy (ΔG) value for all the five simulations. The total binding energies calculated from these MD simulations range from a high of –85 ± 15 kcal mol^−1^ (for the sequences GlcNS6S-IdoA(^2^S_O_)-GlcNAc6S-IdoA2S(^1^C_4_) bound to cluster 1 and IdoA(^1^C_4_)-GlcNAc6S-IdoA2S(^2^S_O_)-GlcNS6S to cluster 5) to low of –117 ± 22 kcal mol^−1^ (for the sequences GlcNAc6S-IdoA2S(^2^S_O_)-GlcNS6S-IdoA2S(^1^C_4_) to cluster 1 and GlcNS6S-IdoA(^2^S_O_)-GlcNS6S-IdoA2S(^2^S_O_) to cluster 5). Several points about these sequences are interesting. The orientation of sequences appears to favour GlcN residues at the non-reducing end suggesting directionality in HS recognition. Although un- or undersulfated GlcN and IdoA residues were favoured in these sequences, despite the presence of fully sulfated GlcNS6S-IdoA2S sequences, the results do not necessarily imply that heparin-like structures are disfavoured. In fact, the sequences appear to be equivalent to heparin-like sequences in HS. Thus, the domain structure known to be present in HS may have special relevance for CXCL13 and its movement along a gradient. Finally, the identification of multiple sequences suggests that nature appears to have engineered hydrogen-bond exchange capability to enable rapid interaction with available HS chains.

### 3.5. CXCL13 dimerization is strongly promoted upon binding to HS

**Figure 6.**
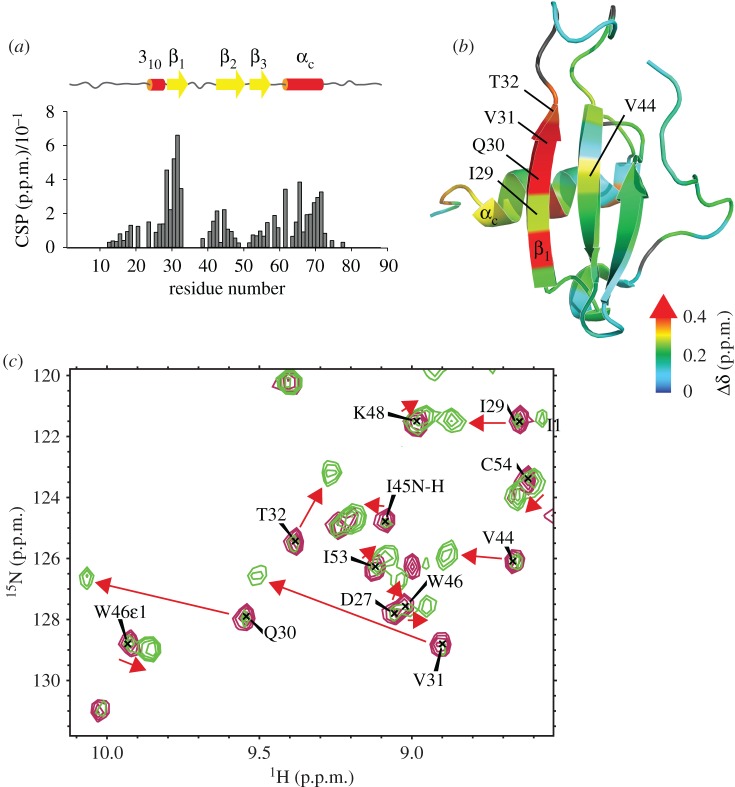
CXCL13 dimerization interface. (*a*) Combined CSPs of CXCL13 amides upon dimerization, assessed using values extracted from the spectrum of Met-CXCL13 at 950 µM or ΔN-CXCL13 at 580 µM. Secondary structures are displayed as thick yellow arrow for β-strand and red cylinder for helix. (*b*) CSP values mapped over the CXCL13 structure. The most affected residues include the β1 strand and the end of the α-helix, localized at the canonical dimerization interface for CXC-chemokine. (*c*) HS binding-triggers CXCL13 dimerization. The overlaid spectra of ΔN-CXCL13 at 50 µM without (purple) or with 50 µM of dp4 (green) are centred on the β-sheet area of 2D [^15^N,^1^H]-correlation spectra. CXCL13 dimerization occurs at the slow-exchange limit compared with chemical shift time scale. Consequently, each residues experiencing dimerization have characteristic chemicals shifts for both oligomeric states. The red arrows depict the transition from monomer to dimer characteristic chemical shifts for those particular residues upon addition of dp4.

All chemokines have a clear structural basis for dimerization with either the N-terminus or the β-sheet providing a scaffold for quaternary interactions [23]. In the latter case, which is a characteristic of the CXC family, the dimer interface arises from anti-parallel association of the first β-strand of each monomer, thus creating a large six stranded β-sheet overlaid by the α-helices. Two populations were simultaneously observed on ΔN-CXCL13 HSQC spectra, and increasing the protein concentration revealed the presence of an oligomeric equilibrium in slow-exchange compared with chemical shift (electronic supplementary material, figure S7). By recording spectra at different concentrations of CXCL13 and measuring the T1/T2 ratios, MWs of 10.5 ± 0.7 kDa and 18.7 ± 1.5 kDa were estimated, indicating that CXCL13 exists in equilibrium between monomer and dimer forms. Changes in CXCL13 chemical shifts upon dimerization provide unambiguous indication on the dimerization interface (figure 6*a*,*b*) and revealed a canonical CXC-type dimer, involving residues of the β1 strand (from Asp27 to Thr33) and residues localized at the end (Leu65, His68, Val69, Gln70 and Ser71) and downstream (Gln70 and Ser71) of the α-helix. The perturbations of the β1 strand residues arise from the anti-parallel association of both protomer β-sheet, whereas the perturbations of the α-helix residues result from opposite β-sheet covering. Moreover, the increase of both NOE number and intensity for Gln70 and Ser71 suggest diminished dynamics and higher conformational restriction. Using peak intensity ratio for residues best sensing dimerization (e.g. Arg28, Ile29 and Thr32) an equilibrium dissociation constant of 0.3 ± 0.1 mM was calculated for dimerization.

In the presence of dp4, the dimer-specific peaks experienced drastic signal increase, revealing the dimerization of the protein (figure 6*c*). Thanks to the slow exchange rate between monomer and dimer forms of CXCL13, we were able to follow chemical shift perturbation upon dp4 binding for both oligomeric forms. It appears that the same set of residues are involved in binding for both monomer and dimer forms. Taking into account that the HS binding cluster 1 of CXCL13 (α-helix) is close to the dimer interface, we hypothesized that a dp4 could stabilize the dimer state by interacting with both α-helices of the dimer.

**Figure 7.**
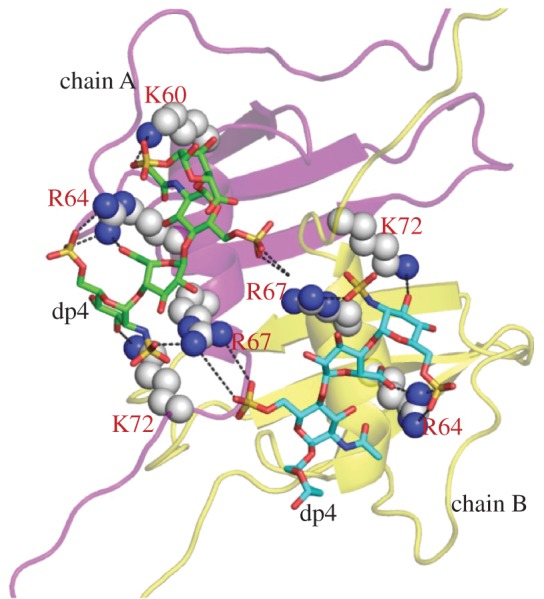
Inter-complex interaction of HS dp4 and CXCL13 homodimer. The interaction of two dp4 molecules binding to two chains of CXCL13 in a symmetrical manner. The inter-molecular interaction shows that both chains form a strong hydrogen-bond interaction with residues Lys60, Arg64, Arg67 and Lys72, respectively (black dotted lines).

To assess this hypothesis, a model of CXCL13 homodimer was calculated by protein–protein docking using HADDOCK (electronic supplementary material, figure S8). Using the CVLS strategy, the top 25 ranked dp4 sequences bound to CXCL13 monomer at binding site I (α-helix) was applied to CXCL13 dimer. Each sequence was docked in triplicate to CXCL13 homodimer for 100 GA runs and 100 000 iterations. Of the 25 sequences, only one dp4 sequence (GlcNS6S-IdoA(^2^S_O_)-GlcNAc6S-IdoA2*S*(^1^C_4_)) bound to CXCL13 dimer with high consistency (RMSD < 2.5 Å). Assessing the inter-molecular hydrogen bonding between the chemokine dimer and the bound oligosaccharide showed strong hydrogen-bond interactions involving residues Lys60, Arg64, Arg67 and Lys72 of Chain A, and Arg67 of Chain B. Of these, the interaction with Arg67 of both chains is likely to be critical. If two dp4 molecules bind in a symmetrically identical manner to the binding site 1 of CXCL13, then multiple inter-complex interactions, as shown in figure 7, would help stabilize CXCL13 homodimer, in agreement with the dp4-induced dimer stabilization observed experimentally.

### 3.6. The HS and the receptor binding sites of CXCL13 are distinct

**Figure 8.**
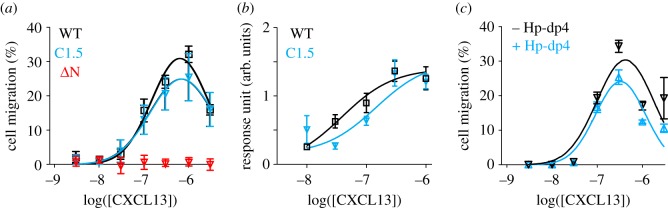
Cell signalling induced by wt and mutant-CXCL13. (*a*) Dose-dependent CXCL13-induced chemotaxis of wt- (black squares), C1.5- (blue triangles) and ΔN- (red triangles) CXCL13. (*b*) Intracellular calcium mobilization induced by wt- (black squares) and C1.5- (blue triangles) CXCL13 on CXCR5-transfected CHO 677 cells. (*c*) Dose-dependent CXCL13-induced chemotaxis of CXCL13 (black triangles) or CXCL13–dp4 (blue triangles). The percentages of migrated splenocytes over the total number of cells or the level of calcium intakes (arbitrary unit) are presented as the mean ± s.d. of three independent experiments.

Although HS binding is well known to regulate chemokine function, the mechanism underlying this control is unclear, notably because in many cases the relationship existing between HS and receptor binding sites has been poorly investigated. We first investigated whether the CXCL13 mutants displayed a reduced ability to bind to cell surface HS. For that purpose, wt- or mutant-CXCL13, featuring a C-terminus S6-tag, were fluorescently labelled using a coenzyme A-Alexa Fluor 488 conjugate (electronic supplementary material, figure S9AB) and incubated with either the CXCR5 negative CHO-K1 or CXCR5/HS negative CHO-pgs-D677 cells. Binding to cell surface monitored by flow cytometry showed that HS binding site (both C1 and C5) mutation of CXCL13 reduced binding to the parental CHO-K1 cell line to the level of that of both wt- and mutant-CXCL13 to the HS deficient CHO-pgs-D677 cells (electronic supplementary material, figure S10). Having demonstrated that the mutants CXCL13 that displayed low (µM) affinity for HS in SPR studies also failed to interact with HS in the context of native cell membrane, while featuring a wt-like structure, we measured their biological activity with chemotaxis and Ca^2+^ mobilization, two hallmarks of chemokine-promoted responses. Our data showed that the wt- and the HS binding disabled CXCL13 induced similar chemotactic activity on CXCR5-positive splenocytes and were also equally functional with regard to Ca^2+^ mobilization performed on CXCR5-transfected CHO cells (electronic supplementary material, figure S11; figure 8*a*,*b*). CXCL13, from which the first nine residues were removed (ΔN-CXCL13) did not induce any cell migration (figure 8*a*), demonstrating the importance of the N-terminal domain for the chemokine bioactivity. As ΔN-CXCL13 binds to HS with an affinity similar to that of the full-length CXCL13, this supports that bioactivity and binding to HS are not overlapping. In agreement with these observations, we also found that free- and dp4 bound-CXCL13 displayed the same biological activity (figure 8*c*), thus further demonstrating that HS and CXCR5 binding sites are distinct.

## 4. Discussion and conclusion

Chemokines, although relatively small-sized proteins, features three important functional interfaces, characterized by increasing level of diversity: the receptor activating domain, which consistently involves the chemokine N-terminal peptide [27], the dimerization domain involving either the β-sheet or the N-terminal strand and the HS binding sites which can occur on up to five different clusters [3]. Depending on whether or not these regions are spatially distinct or overlapping, different situations can theoretically occur: (i) HS-bound chemokines need to be released to interact with their receptors (for example, when the HS and receptor binding sites overlap); (ii) HS can present chemokines in a bound form (the two binding sites are spatially distinct); and (iii) HS modifies the chemokine's presentation to their receptors (for example, it induces structural modifications).

Here, to investigate the mechanism by which HS could regulate CXCL13 activity, we solved its solution structure and characterized its dimerization interface and HS binding sites. We report that CXCL13 has a canonical chemokine core structure, to which is appended an unusual specific 19 amino acid long domain, downstream of the C-terminal α-helix that remains unstructured. Such C-terminal extensions, comprising at least 10 residues, are only found in a small subset of chemokines (CXCL9, CXCL12γ, CCL16, CCL21, CCL24, CCL25 and CCL28). Surprisingly, we observed that *E. coli*-expressed CXCL13, featuring an additional N-terminal Met residue, has its N-terminal sequence folded over the first β-strand of the chemokine, a feature that was also reported for the initiating Met-containing human CXCL13 (pdb 4ZAI). We report that such a folding, which would not be compatible with the receptor activation mechanism and would prevent dimerization, is specifically driven by the N-terminal Met, which is absent in the original eukaryotic protein.

Two regions of CXCL13 contribute to HS binding. The first one, in common with many CXC chemokines, is found in the α-helix. In contrast to many other CXC chemokines, however, the second one is not present within the 40's loop. Although the loop structure of CXCL13 is similar to that of both CXC- and CC-chemokines, for which HS binding has been shown to involve this particular site (i.e. CXCL1, CXCL2, CXCL4, CCL4 and CCL5), it is worth noting that it features a Met49 that, in the five other chemokines, aligned with an asparagine or a basic residue. The presence of this bulky residue, localized within the basic sequence of the CXCL13 40s loop (electronic supplementary material, figure S12), is thus likely to hinder access to HS. For CXCL13, the second HS binding cluster was found in the C-terminal extension in the form of a small basic sequence (KRR) that is well conserved throughout evolution (KRK in human). We showed that the CXCL13-HS interaction was characterized by an affinity of approximately 20 nM, mostly contributed by the cluster 1 α-helix HS binding site. Mutations of the C-terminal basic cluster decreased the affinity to 80 nM, without modifying the rate at which the protein dissociates from HS. Such a mechanism has been reported for IFNγ, an HS binding cytokine [28], for which a basic cluster (RGRR) also present at the extremity of the unstructured C-terminal sequence was shown to enhance the association rate of the binding, without affecting the stability of the formed complex [20]. Interestingly, the opposite situation was reported for CXCL12γ. This chemokine, features two HS binding domains, located within the first β-strand and within the disordered 30 amino acid long C-terminus. Kinetic analysis showed that this latter domain functions by stabilizing the CXCL12γ-HS complexes, giving rise to a sub nanomolar affinity, without significantly affecting the association rate constant of the binding reaction [29]. Consistently with this very high affinity, CXCL12γ is essentially found in an HS-bound form [30], and recent work showed that it induced robust CXCR4-dependent migration only when presented to its receptor in a HS-dependent manner [31]. Together, these data show the interest of characterizing the kinetic aspects, beyond equilibrium affinity, of HS binding. Up to now, among the C-terminal extended chemokines, this has only been investigated for CXCL12γ and CXCL13. For CCL24, CCL25 and CCL28, which all bind to HS [32–34], the HS binding sites have not been formally identified; therefore, the role of the C-terminal domain in this process remains unknown. The C-terminal peptide of CXCL9 displays strong affinity for heparin [35], but its contribution in the context of the full-length protein has not been reported. Finally, it has been shown that CXCL16, following MMP-12 mediated processing, which remove 7 and 20 residues from the N- and C-terminal domains, respectively, features an enhanced affinity for HS, presumably through exposing a previously buried binding site [36].

Recent *in vivo* studies strongly supported a role for HS in establishing chemokine gradients that are more robust and spatio-temporally stable than soluble ones [4,37]. The fact that both CXCL12 and CXCL13 can be functionally presented to their signalling receptor supports the view that binding to HS can contribute to their localization within tissues. Given the kinetic differences that characterized CXCL13 and CXCL12γ, it would be interesting to know whether the transient or the stable nature of the complex they form with HS contributes to their respective distribution and biological function within germinal centres. Using a model of CXCL8-induced leucocyte migration, it has been observed that, in addition to allowing chemokine to form optimal gradient, chemokine immobilization restricts cell motility near the source, thus mediating both recruitment and retention of cells at sites of infection [4]. Similarly, truncation of the CCL21 C-terminal extension, importantly reduced HS binding and converted the matrix immobilized form of the chemokine which caused localized and random movement of cells, into a soluble form that promoted chemotactic movement [37].

Dimerization also represents a mechanism by which chemokine can be locally concentrated [38]. According to our data, with a dimerization *K*_D_ of 0.3 mM, CXCL13 is a monomer at physiological concentrations. The dimerization of CXCL13, which can be observed at very high concentration, involved the anti-parallel association of both protomer β-sheets. Surprisingly, the time scale of dimerization is slow compared with chemical shift time scale, which is uncommon for millimolar-range affinity dimer and suggests that the association rate is lower than expected. The low propensity to form dimer might be a characteristic of chemokine featuring long C-terminal extensions, such as CCL21 [39] and CCL24 [40], which remain monomeric at 0.5 and 1 mM, respectively. This could be due to the flexibility of the C-terminal domain, sampling a large space around the protein, hence partially hampering the close association of the two monomers. Analysing the dimerization propensity of CXCL9, CXCL16, CCL25 and CCL28—which structures are still unknown—would help confirm this hypothesis. Regarding CXCL13, our data demonstrated that its dimerization is nevertheless induced upon binding to HS, as short as dp4. Such dimer, which leaves the N-terminus and N-loop exposed on the chemokine surface, can activate the receptor and could represent a functional unit which local concentration would be increased by HS binding. The physiological relevance of this possibility remains nevertheless to be investigated.

As mentioned earlier, CXCL13 importantly regulates the migration and positioning of T- and B-lymphocytes in secondary lymphoid organs where it cooperates critically with CXCL12 in the formation of germinal centres. While CXCL12 is abundantly present in the dark zone where B cells migrate and undergo somatic hypermutations of their antibody variable genes, CXCL13 directs them to the light zone where are also found T helper lymphocytes promoting their antigen-driven selection. An open question is whether CXCL12 and CXCL13 recognize specific HS sequences that would explain their selective retention in distinct areas of the germinal centre. In that context, we have used computational techniques to gain a better understanding of HS binding to CXCL13. The top 25 ranked sequences of dp4 bound to CXCL13 monomer at binding site I (α-helix) were used for studying the interaction of CXCL13 dimer with dp4. Interestingly, of the 25 sequences only one bound to CXCL13 dimer with high consistency. Computational studies indicate that significant specificity of recognition arises from hydrogen bonding with several residues, of which Arg67 is more important. Although it is still difficult to ascribe this selectivity of recognition to the differential accumulation of chemokines in tissue, the results indicate the possibility that the known spatial and temporal expression of HS in tissues may lead to differential accumulation of HS-binding proteins. Importantly, our work also reports the engineering of mutant-CXCL13 displaying a strongly impaired ability to bind HS, while NMR analysis showed that their overall structures were not affected, and functional studies validated that these mutations do not affect their biological activity. The fact that the HS site can be manipulated without affecting receptor binding and activation should permit the development of animal models expressing a non-HS binding mutant of the chemokine, but remaining fully functional. Thus, using the strategy that we previously developed for CXCL12, this should permit the unambiguous evaluation of the contribution of HS-mediated immobilization to the biological activity of this chemokine.

## Supplementary Material

Supplementary methods, references and figures

## Data accessibility

Chemokine structures have been deposited in the PDB (Protein Data Bank) under PDB ID: 5L7M and 5IZB. Datasets supporting this article have also been uploaded as part of the electronic supplementary material.
