# Supplementary material for: Solution structure of CXCL13 and heparan sulfate binding show that GAG binding site and cellular signalling rely on distinct domains
Source: Open Biol. 2017 Oct 25;7(10):170133. doi: 10.1098/rsob.170133 (PMC5666081; doi:10.1098/rsob.170133)
Supplement: Supplementary methods, references and figures [file rsob170133supp1.pdf]

SUPPLEMENTARY METHODS, REFERENCES AND FIGURES FOR:

Solution structure of CXCL13 and heparan sulfate binding show that GAG binding site and cellular signalling rely on distinct domains

Yoan R. Monneau[1‡], Lingjie Luo[2‡], Nehru Viji Sankaranarayanan[3,4], Balaji Nagarajan[3,4], Romain R. Vivès[1], Françoise Baleux[5], Umesh R. Desai[3,4], Fernando Arenzana-Seidedos[2] and Hugues Lortat-Jacob[1*]

METHODS

Wild type and mutant CXCL13 expression and purification

CXCL13 was cloned into a pET-17b plasmid, resulting in a protein construct of 89 amino acids, including the initiating methionine (9.9 kDa). All mutations were incorporated in Met-CXCL13 gene by one-step site-directed mutagenesis as previously described [1]. Briefly, mutations-containing primers initiated the amplification of the whole plasmid and the products were treated by Dpn1 1 h at 37°C prior to transformation of competent cells. All CXCL13 proteins were expressed in *E. coli* BL21-codonPlus strain (Agilent), grown in 1 L of M9 minimum media supplemented with $^{15}NH_4Cl$ (1 g.L^{-1}) and either $^{12}C_6$-glucose or $^{13}C_6$-glucose (2 g.L^{-1}). Cell growth at 37°C preceded induction with 0.5 mM of isopropyl β-D-thiogalactopyranoside at OD_{600} = 0.8 and the cells were harvested 3 hours later by centrifugation (15 min at 7,000g). The pellet was resuspended in 50 mM Tris and 1 M NaCl at pH 8 (buffer 1) prior to extensive bacteria lysis by sonication and centrifugation (22 min at 50,000g). The pellet was washed four times by sequential solubilisation and centrifugation steps with buffer 1, supplemented with 2 M urea with or without 5% (v/v) Triton X100 for the first and the second wash step, respectively. The washed pellet was then treated with 10 mL of 6 M Guanidine HCl and 20 mM DTT for 1.5 h at 37°C under shaking and cleared by centrifugation (10 min at 16,000g) prior to loading into Hi-load superdex S75 column (GE healthcare) equilibrated with the running buffer containing 7 M urea, 1 M NaCl, 50 mM potassium phosphate and 2 mM β-mercaptoethanol at pH 6. To proceed with

CXCL13 refolding, the elution fractions were diluted seven-times in refolding buffer (50 mM Tris at pH 8) and kept at 4°C overnight under gentle rocking. Four times dilution in refolding buffer preceded the loading into FastFlow SP sepharose ion exchange column (GE healthcare) equilibrated with a 50 mM potassium phosphate buffer at pH 6. Elution was performed under NaCl gradient (from 0 to 1 M) for 30 min at 2 $mL.min^{-1}$. Elution fractions were concentrated up to reach 3 mL volume (MWCO 3000 Millipore Centricon) prior to loading into Hi-load superdex S75 column (GE healthcare) equilibrated with the running buffer containing 20 mM potassium phosphate and 100 mM NaCl at pH 6. Elution fractions were concentrated up to reach 1 mL volume and the final concentration was measured by absorbance at 280 nm based on a molar extinction coefficient of 12,740 $M^{-1}.cm^{-1}$ (http://www.expasy.ch).. CXCL13 construct, including an enterokinase cleavage site, was process similarly. To eliminate the -1 Met residue, enterokinase digestion (2U Sigma) was performed in 500 µL of 50 mM TriB pH 8 for 3h at room temperature with, prior to loading into the Hi-load sephadex S75 column. The digestion was validated by N-terminal amino acids sequencing. All CXCL13 samples were then stored at -80°C.

NMR spectroscopy

NMR signal assignment of wt- and Met- CXCL13 was performed on 200 µM protein samples containing 20 mM phosphate buffer (pH 6) and 100 mM NaCl. Spectra were recorded at 298 K using Bruker AvanceIII 600, 700, 850 or 950 MHz NMR spectrometers all equipped with cryogenic probes. Data processing with NMRPipe [2] preceded analysis with NMRFAM-SPARKY 1.2 [3]. Backbone and aliphatic side-chain assignments were obtained from 2D ^{15}N-HSQC, 3D HNCACB, 3D CBCA(CO)NH, 3D HNCA, 3D HN(CO)CA, 3D HNCO, 3D HCACO spectra recorded on 5% $^{2}H_2O$ sample and 2D ^{13}C-HSQC, 3D HC(C)H-TOCSY, 3D (H)CCH-TOCSY spectra recorded on 99% $^{2}H_2O$ sample. Aromatic side-chain assignments were performed on 99% $^{2}H_2O$ sample with 2D ^{13}C-HSQC, 2D ^{1}H-TOCSY, 2D DQF-COSY, and 2D (HB) CB(CGCD)HD. The Met-C_ε resonances were based on 2D ^{13}C-HSQC and 3D ^{13}C-NOESY spectra. Stereospecific assignments of Val and Leu methyl were obtained with the labeling method previously described [4] along with the recording of a high resolution 2D ^{13}C-HSQC.

The combined chemical shift perturbation $\Delta\partial_i^{NH}$ of the ith residue upon dimer association or heparin binding was calculated using equation (1), as described [5].

(1): $\Delta\partial_i^{NH} = \sqrt{\left[\delta_i^H - \delta_{i,ref}^H\right]^2 + \left[0.14(\delta_i^N - \delta_{i,ref}^N)\right]^2}$

with δ_i^H and δ_i^N, the ^1H and ^{15}N chemical shifts, respectively, of the ith residue; $\delta_{i,ref}^H$ and $\delta_{i,ref}^N$, the ^1H and ^{15}N chemical shifts, respectively, of the ith residue in the reference spectrum.

The equilibrium thermodynamic constants of dimerization were calculated from NMR signal of proper residues (see result part) as described, using equation (2) for the monomer-dimer association.

(2): $K_D = \frac{2[P]_{tot}\left({}^{a_M}/_{a_D}\right)^2}{1+{}^{a_M}/_{a_D}}$

with K_D, the thermodynamic dissociation constant; $[P]_{tot}$, the total concentration of CXCL13; a_M and a_D, the NMR signal of either monomer- or dimer-belonging residue amide, respectively. The thermodynamic constants are presented as the average over all calculated ones (one per residue of interest) ± standard deviation of mean.

The longitudinal (T1) and transversal (T2) ^{15}N relaxation times and the heteronuclear (^1H)-^{15}N steady-state NOE were measured using previously published methods and pulse sequences [6]. The set of times were 0 to 1.5 s and 0 to 0.6 s for T1 and T2 measurements, respectively, and the saturation time was 3 s for NOE enhancement experiments. The recovery delays were set to 3 s and 5 s for T1/T2 and {(^1H)-^{15}N} NOE measurements, respectively, to ensure that all ^{15}N magnetization returns to equilibrium and to lower intensity decrease due to amide proton exchange with saturated water. The data were recorded at 291 K on Met-CXCL13 at 680 µM and at 298 K on ΔN-CXCL13 at 750 µM.

The molecular weight has been estimated using NMR approach previously described [7]. Briefly, the tumbling correlation time (τ_c) is calculated based on the ratio T1/T2 according to the equation 8 of Kay et al. [8] and considering the slow molecular motion limit (τ_c >> 0.5 ns) and the use of high frequency spectrometer (> 500 MHz). The τ_c can then be defined for secondary structure-localized residues as

(3): $\tau_c \approx \frac{1}{4\pi\nu_N}\sqrt{6\frac{T1}{T2} - 7}$

with ν_N, the ^{15}N Larmor frequency (Hz). Considering the protein behaves like a hydrated sphere and according to the Stoke-Einstein law, the tumbling correlation time is also defined as

(4): $\tau_c \approx (\overline{V}_p + \alpha\overline{V}_{H2O})\eta\frac{MW}{RT} \approx 0.38 \times 10^{-12}MW$

with MW, the protein molecular weight; R, the gas constant; η, the dynamic viscosity of water; and T, the absolute temperature. This latter equation was simplified considering the partial specific protein volume, $\overline{V}_p$ = 0.73 $cm^3.g^{-1}$ and α = 0.3 g of bound water per gram of protein; 1 $cm^3.g^{-1}$ and 0.9 cP for the partial specific volume ($\overline{V}_{H2O}$) and the dynamic viscosity of water. According to the plot of rotational correlation time versus protein molecular weight for known monomeric NESG (North East Structural Genomics Consortium) targets of ranging size, τ_c/MW was experimentally measured as $0.6x10^{-12}$ $mol.g^{-1}$ [7]. Using the experimentally determined τ_c/MW ratio and the first equation, the molecular weights of both oligomeric states of CXCL13 were estimated.

Molecular Dynamics Studies

Wt-CXCL13 with dp4 bound at both α-helical region cluster 1 and C-terminal region cluster 5 from the CVLS method was used as the initial structure for unrestrained constant temperature and pressure MD simulation. Each co-complex (wt-CXCL13–dp4) involving different combination of dp4 sequences bound at the binding sites were systematically prepared using Leap module of AMBER14 as follows. Amber-ff12SB force field and GLYCAM_06j-1 force field parameters were used for protein and ligand preparation respectively [9]. The net charge of each co-complex was neutralized to zero by adding appropriate number of Na+/Cl- counter ions, then centred in a three-point water (TIP3P) molecule box with a minimum distance of 12 Å between the walls to any atom in the complex. The system was then relaxed to a minimum state energy in two steps: i) the solute atoms were restrained with a force constant of 100 kcal/(mol. Å2) and the solvent molecules relaxed using 500 cycles of steepest descent and 2000 cycles of conjugate gradient method; ii) the whole system was relaxed using conjugate gradient minimization of 2500 cycles without any restraints. Following this each co-

complex was brought to desired temperature and pressure (NPT) and further equilibrated for 1 ns. The MD production run was performed in NPT ensemble with the integration time step of 1 fs. Bonds involving hydrogen atoms were constrained using SHAKE algorithm. Maxwell distribution was used to assign the initial velocities and the total trajectory of each co-complex was computed for 50 ns.

Equilibration and the simulation processes were validated using physical observables of the system including potential/kinetic energies as well as temperature and pressure. The sugar puckering for the IdoAp heterogeneity was restrained at the desired puckering ($^{2}S_{O}/^{1}C_{4}$) during the entire simulation, while the sugar puckering of GlcNp heterogeneity was stable in $^{4}C_{1}$ Conformations throughout the simulation.

Binding free energy calculation of each co-complex was computed using post-processing MM/PB(GB)SA method [10] from the trajectories. MMGBSA employed single residue energy decomposition (SRED) to estimate the energy contribution of the single receptor residue responsible to the bound state. Both the energy calculations were performed with the default parameter settings by employing python version of MM/PB(GB)SA module from Amber Tools13[11]. These energy calculations were performed for the last 40 ns from each simulation with the structures from every 10 ps (a total 4,000 structure frames).

Preparation and characterization of fluorescently labeled CXCL13

S6-(GDSLSWLLRLLN) tagged wt- or mutant- CXCL13 were prepared by chemical synthesis (ALMAC, East Lothian, UK) and fluorescently labeled essentially as described [12]. The labeling reagent was prepared by stirring in the dark, 1 h at room temperature, a mixture made of 1.4 µmole of Alexa Fluor 488 C5-maleimide (Life technologies) in 150 µl DMSO and 2.8 µmole of Co-A lithium salt dissolved in 750 µl of 50 mM sodium phosphate buffer pH 7.2. The coenzyme A (CoA)-Alexa Fluor 488 reagent was then purified on a 5 µm C18 300 Å Kromasil column (10 x 250 mm) using a 5-25% linear gradient of acetonitrile in 10 mM triethylamine acetate buffer over 20 min (6 ml/min flow rate, detection at 230 nm). Retention time: 9 min. Isolated yield: 90% based on 488 nm absorption (72000 cm^{-1} M). Molecular weight was controlled by HR-Mass (Q-TOF micro, Waters, collision: 5V, cone: 20V) using negative mode. Expected monoisotopic mass $[M-H]^{-}$ for $C_{51}H_{62}N_{11}O_{22}S_{3}P_{3}$: 1464.2063. Found: 1464.2739. The reagent was lyophilized, suspended in water at 0.6 mM and stored at -20°C in the dark.

The labelling reaction was performed by mixing the S6-tagged wt-CXCL13, the CoA-Alexa Fluor 488 reagent, and the Sfp synthase (New England Biolabs) at 10 µM, 19 µM, and 1 µM respectively, in 50 mM HEPES, 10 mM MgCl2, pH 7.2. The reaction was incubated in the dark at room temperature for 30 min, and the Alexa Fluor 488 S6-tagged wt-CXCL13 was purified on a 5 µm C18 300 Å Kromasil column (10 x 250 mm) using a 0-50 % linear gradient of acetonitrile in 0.08% aqueous TFA over 50 min (6ml/min flow rate, detection at 230 nm). The retention time was 37 min, the isolated yield, 65% (quantified by amino acid analysis on Hitachi L8800 apparatus) and the final purity, as controlled by analytical RP-HPLC (AERIS Peptide, 3.6 µm XB-C18, 2.1 x 100 mm, 5-25 % linear gradient with same eluents as above, 0.350 ml/min flow rate, RT=13,4 min), was 93 % (Figure S6A). The labelled protein was lyophilized following purification and the labelling validated by ESI-TOF MS (Figure 6B).

Preparation of heparin derived oligosaccharides and biotinilated heparan sulfate

Porcine mucosal heparin was digested with heparinase I (8 mU/ml) in 0.1 mg/ml BSA, 2 mM $CaCl_2$, 50 mM NaCl and 5 mM Tris buffer pH 7.5 for 50 h at 25 °C. The enzymatic reaction was stopped by heating the digest at 100 °C for 5 min. The digestion mixture was resolved from di-(dp2) to octa-(dp18) decasaccharide using a Bio-Gel P-10 column, equilibrated with 0.25 M NaCl and run at 1 mL/min. To ensure homogeneity, only the top fractions of each peak were pooled, and each isolated fraction was re-chromatographed on a gel filtration column to further eliminate possible contamination. Samples were dialyzed against distilled water and quantified. Heparan sulfate (HS) at 1 mM in phosphate buffer saline (PBS) was biotinylated at its reducing end for 24 h at room temperature with 10 mM biotin-LC-hydrazide [13]. Extensive dialysis of the mixture against H_2O was performed to remove unreacted biotin prior to freeze-drying.

Equilibrium dissociation constant determination

K_D was determined by fitting the maximal responses for each CXCL13 concentration against the Hill equation

(5): $\Delta \mathrm{RU}_{SS}^{i} = \frac{\Delta \mathrm{RU}_{max}}{1+\left(\frac{\mathrm{K_d}}{[CXCL13]_i}\right)^n}$

with $\Delta\mathrm{RU}_{SS}^{i}$, the signal when the steady state is reached for the concentration of ligand $[CXCL13]_i$; $\Delta\mathrm{RU}_{max}$, the maximal signal obtained when all binding site are occupied; and n, the Hill coefficient, using the least squares method (Levenberg-Marquardt algorithm provided by Igor Pro version 6.03) with the three variables n, K_D, $\Delta\mathrm{RU}_{max}$ as free fit parameters.

CXCL13 cell surface binding assays

Cell surface binding assays were conducted using fluorochrome-coupled chemokine. CXCL13-Alexa488 were diluted in 200 µL 20 mM Hepes, 1% BSA, RPMI1640 prior to incubation at 4°C for 90 min at the indicated concentration with 5×10^5 of either parental CHO-K1 or HS-deficient CHO-pgs-D677 cells. Unbound chemokine was washed out with RPMI 1640 buffer supplemented with 20 mM Hepes and 1% BSA. After washing, cells were suspended with PBS buffer supplemented with 0.5% BSA and 0,1% sodium azide, and remaining cell-bound chemokine was monitored by flow cytometry (FACScantoTMII Flow Cytometer, BD Bioscience, USA).

Figure S1

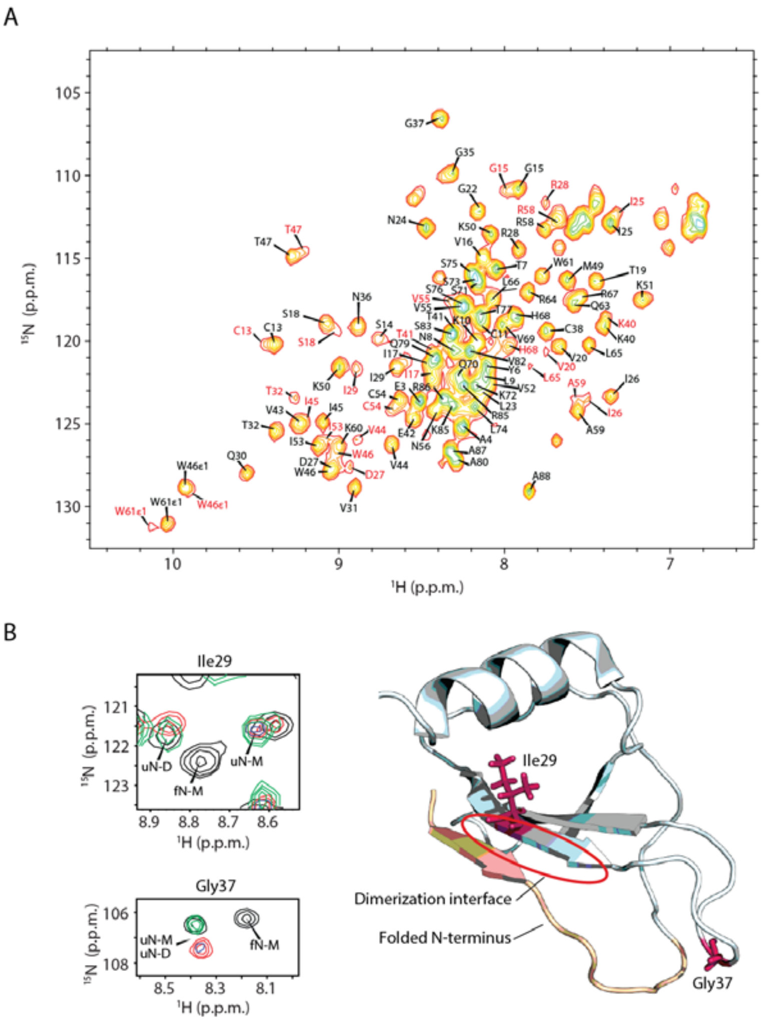

A: The 2D [^{15}N,^{1}H]-correlation spectrum of wt-CXCL13 at 75 µM is shown with monomer- and dimer- specific signals displayed in black and red, respectively. B: The overlaid 2D [^{15}N,^{1}H]-correlation spectra of wt-CXCL13 at 950 µM (black), met-CXCL13 at 75 µM (green) and ΔN-CXCL13 at 56 µM (blue) or 560 µM (red) are shown centered on Ile29 and Gly37 (‘fN-M’, ‘uN-M’ and ‘uN-D’ state for folded N-terminus monomer, unfolded N-terminus monomer and unfolded N-terminus dimer respectively). The Met-CXCL13 structure featuring the folded N-terminus is displayed to show that Ile29 can probe both N-terminal folding and dimerization and Gly37 can probe only the N-terminal folding. The wt-CXCL13 does not display any characteristic resonances of the folded N-terminus.

Figure S2

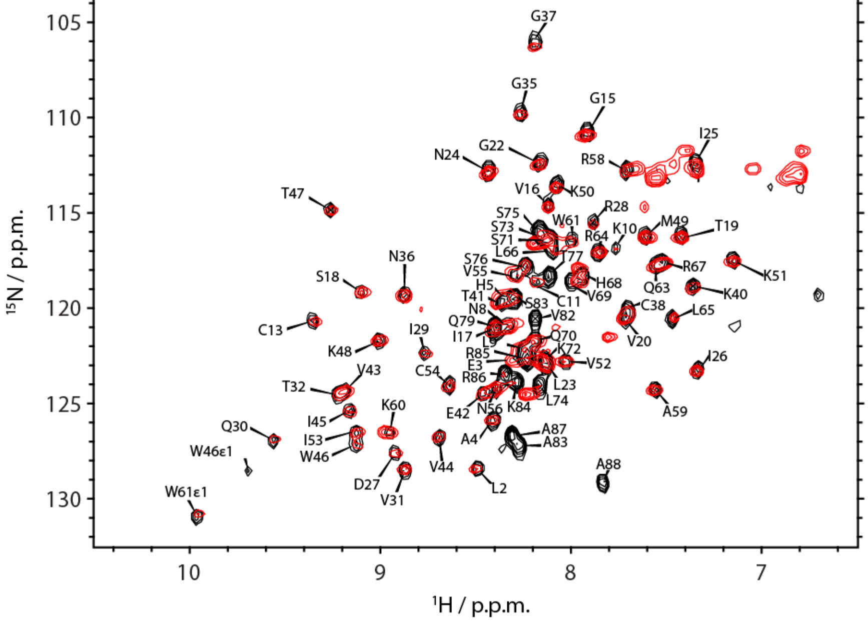

The CXCL13 C-terminus does not interact with the core domain of the protein. The 2D [^{15}N,^{1}H]-correlation spectra of Met-CXCL13 at 37 μM and Met-CXCL13-ΔC (from S75 to A88) at 10 μM are overlaid. The only residues of Met-CXCL13-ΔC that experience chemical shift changes, compared to the full length chemokine, are those localized just upstream of the introduced stop codon (from H68 to L74).

Figure S3

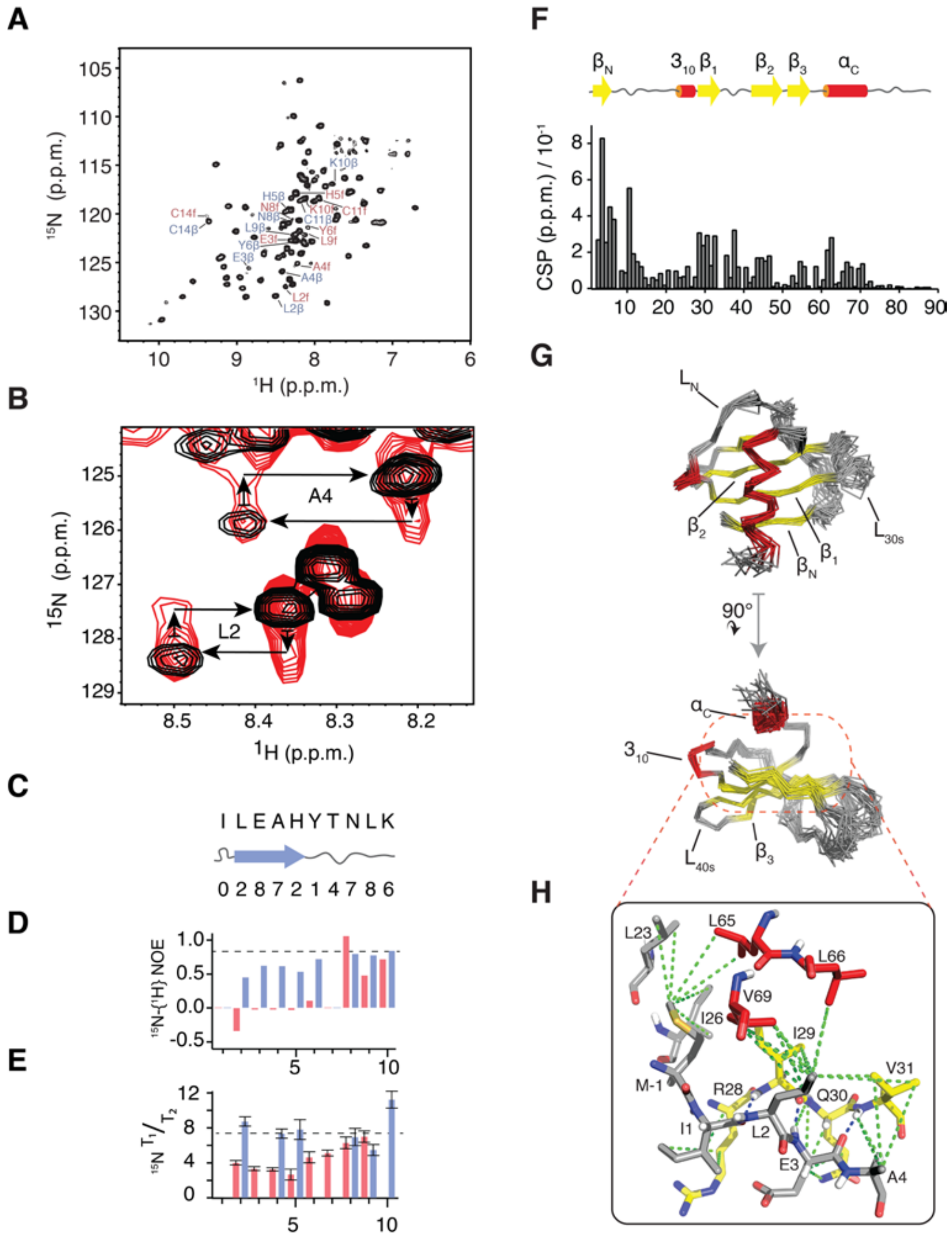

Structure and dynamics analysis of murine Met-CXCL13. **A:** The 2D [^{15}N,^{1}H] correlation spectrum was recorded at 298K on 100 µM U-[^{15}N,^{1}H]-Met-CXCL13 sample (construct including initiating methionine), in 20 mM KP (pH 6) and 100 mM NaCl. The Met-CXCL13 N-terminal residues (Ile1 to Lys10) feature two sets of

simultaneously observable NMR signals within the $^{15}N^{1}H$-HSQC spectra, revealing the existence of two structural states in slow-exchange compared to NMR time-scale. The assignment of N-terminal residues (Leu2 to Cys14) is displayed for either the flexible conformer (in red with 'f' mark) or β-strand-including conformer (in blue with 'β' mark). According to the signal distribution, the structured state is overpopulated compared to the flexible one. **B**: The overlaid ZZ exchange spectra [6] was recorded on Met-CXCL13 at 820 μM with either a mixing time of 0 s (in black) or 1 s (in red). The arrows display the magnetization exchange for the Leu2 and the Ala4. Magnetization exchange experiment further demonstrates exchange between two conformational states. **C**: The secondary structure prediction issued by TALOS-N is shown only for the folded state and consists of a β-strand (blue arrow) from Leu2 to His5, whereas the other state is predicted entirely flexible. The primary structure of CXCL13 is shown above the secondary structure scheme and the issued confidence levels are notified below, which are particularly high for Glu3 and Ala4. **D**: The ^{1}H-$\{^{15}N\}$ NOEs, and **E**: the relaxation times ratios (T1/T2) have been measured at 291K on Met-CXCL13 at 680 μM for each residues belonging to both folded (blue bars) and unfolded states (red bars). The dashed line highlights the average values for the core protein residues. **F**: The combined chemical shift perturbations of Met-CXCL13 amides upon release of the N-terminal domain were assessed using values of both N-terminal states directly obtained from the wild-type spectrum for the first fourteen residues, and using values obtained from the wild type Met-CXCL13 spectrum and the ΔN-CXCL13 spectrum for the rest of the protein. Altogether, these latter results strongly support that one state adopts a β-strand conformation from Leu2 to His5 and interacts with the β1-strand and, to a lesser extent, the C-terminal α-helix, while the second state features conformationally dynamic residues. The solution structures of the monomeric Met-CXCL13 featuring either a folded or unfolded N-terminal domain were solved using torsion angle dynamics preceding rotamer refinement in presence of water, along with NOEs-based set of distance constraints extracted from standard 3D heteronuclear-edited-NOESY. The summary of conformationally restricting constraints and structure quality factors is reported in table S1 for the structured N-terminal tail-featuring Met-CXCL13 ensemble, which was deposited in the Protein Data Bank under accession number 5IZB. **G**: The structure ensemble of Met-CXCL13 featuring a folded N-terminal domain is displayed as ribbon. The secondary structure elements, including four β-strands (yellow) and two helices (red), are named according to canonical

chemokine structures. For clarity, the C-terminal tail is not displayed. **H**: The details of the N-terminal domain (grey) interacting with the core protein are presented. The core protein residues, for which dipolar coupling with residues of the N-terminal domain have been recorded (green dashed line), belong to the strand β1 (yellow), the helix 3_{10} (light grey) and the C-terminal helix (red). The three hydrogen bonds between the β1-strand and the βN-strand are also depicted (blue dashed line). The side chain of N-terminal residues, including Met-1, Leu2 and Ala4 are in close contact with hydrophobic, methyl-containing residues such as Leu23, Ile26, Ile29, Val31, Leu65, Leu66 and Val69, as reported by the recorded NOEs (*green dashed lines*). Minimizing the hydrophobic side-chain exposure to water appears to be the main force that drives N-terminal domain folding. We calculated that, in presence of Met-1, the N-terminal folding triggers the burying of 485 Å^2 of solvent accessible nonpolar surface area (ASA-np), while it was 383 Å^2 in its absence. The contribution of the initiating Met is thus around 20 % of the total free energy related to the so-called ‘hydrophobic effect’. Considering that, according to our data and in agreement with the crystal structure of human CXCL13, two hydrogen bonds also participate in the N-terminal stabilization, the relative contribution of the initiating methionine might be lower. However, the absence of initiating methionine is enough to completely disrupt N-terminal interaction with the core protein.

Figure S4

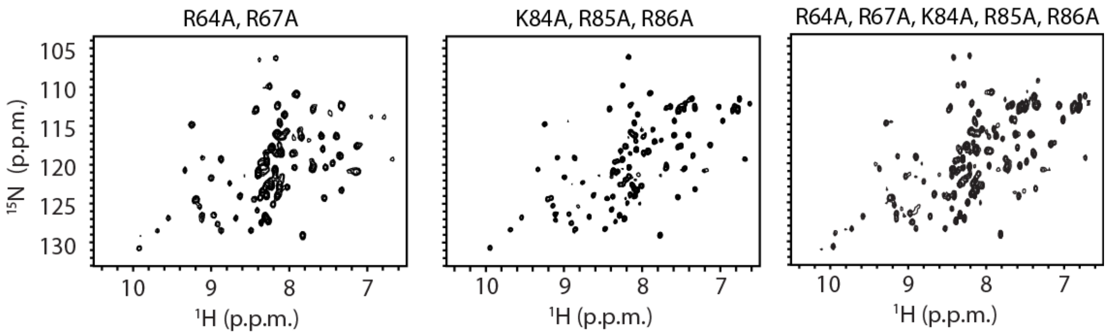

CXCL13 mutants feature structure fingerprint identical to that of the wild type. 2D [^{15}N,^{1}H] correlation spectra were recorded at 298K on the [R64A, R67A] Met-CXCL13 (C1 mutant) at 224 µM, the [K84A, R85A, R86A] Met-CXCL13 (C5 mutant) at 80 µM and the [R64A, R67A, K84A, R85A, R86A] Met-CXCL13 (C1.5 mutant) at 388 µM.

Figure S5

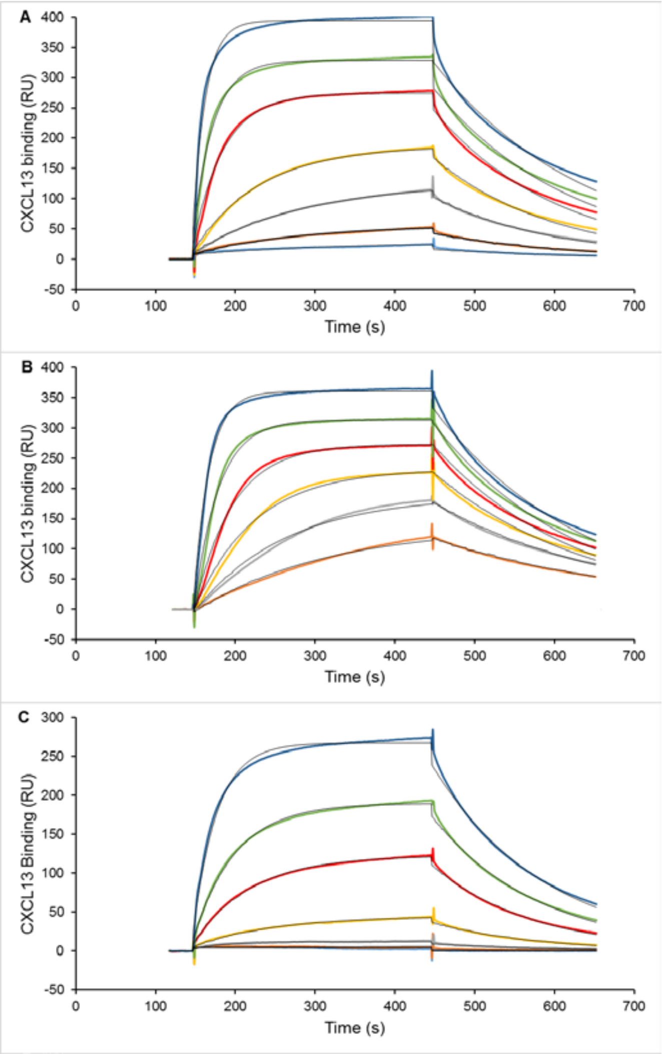

Kinetic analysis of Met-, ΔN- and Met-C5- CXCL13 binding to HS. **A**: Mett-CXCL13, **B**: ΔN- and **C**: Met-C5- CXCL13 were injected over a HS-activated surface at a flow rate of 50 µl/min for 5 min, and the binding signal (in RU) was recorded as a function of time (in s). Each set of sensorgrams was obtained with CXCL13 at (from top to bottom) 140, 93, 62, 41, 28, 18, 12 (Met- and ΔN) and 167, 112, 74, 33, 22, 15 nM (Met-C5-mutant). The association and dissociation phases (coloured traces) were simultaneously fitted to a 1:1 Langmuir binding model, for each CXCL13 concentrations,

and superimposed to the experimental data (black traces). This analysis (performed on the five upper concentrations) returned dissociation rate constants (k_{off}) of 5.8 ± 0.34 x 10^{-3}, 5.3 ± 0.24 x 10^{-3} and 7.5 ± 0.04 x 10^{-3} s^{-1} for the Mett- ΔN- and Met-C5- CXCL13, respectively. The association rate constant (k_{on}) showed increased values with CXCL13 concentrations that were averaged, giving rise to k_{on} of 3.08 ± 0.05 x 10^{5}, 2.7 ± 0.6 x 10^{5} and 9.14 ± 0.58 x 10^{4} M^{-1} s^{-1} for the Mett-, ΔN- and Met-C5-CXCL13 respectively. Presumably, increase in CXCL13 concentration induces dimerization, resulting in a sample that displays a larger HS-binding surface than their corresponding monomer counterpart. These kinetic values should thus be considered as estimates only.

Figure S6

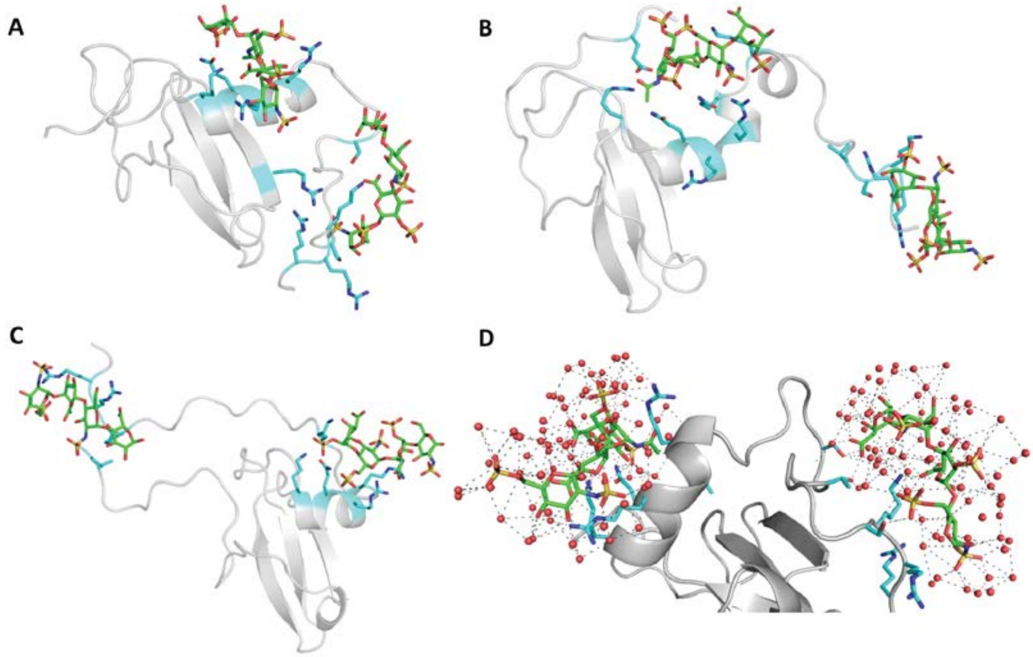

Conformational space exploration of CXCL13 dp4 complex. Conformational sampling of complexes from different simulations are shown to give an overall picture of how the movement of the complex was observed in presence of solvent. A to C shows the different conformational orientation of the complex with the bound tetra saccharide dp4 shown in green colour (sticks) and the interacting residues from Cluster1 and Cluster5 type shown in cyan colour (sticks). (D) The bed of water molecules forming a network around the binding region of CXCL13 dp4 is also represented, as the direct bridging water molecules between CXCL13 residues and dp4 atoms. All the water molecules are shown in red (spheres), dp4 in green (sticks) and the interactions are shown in black (lines).

Figure S7

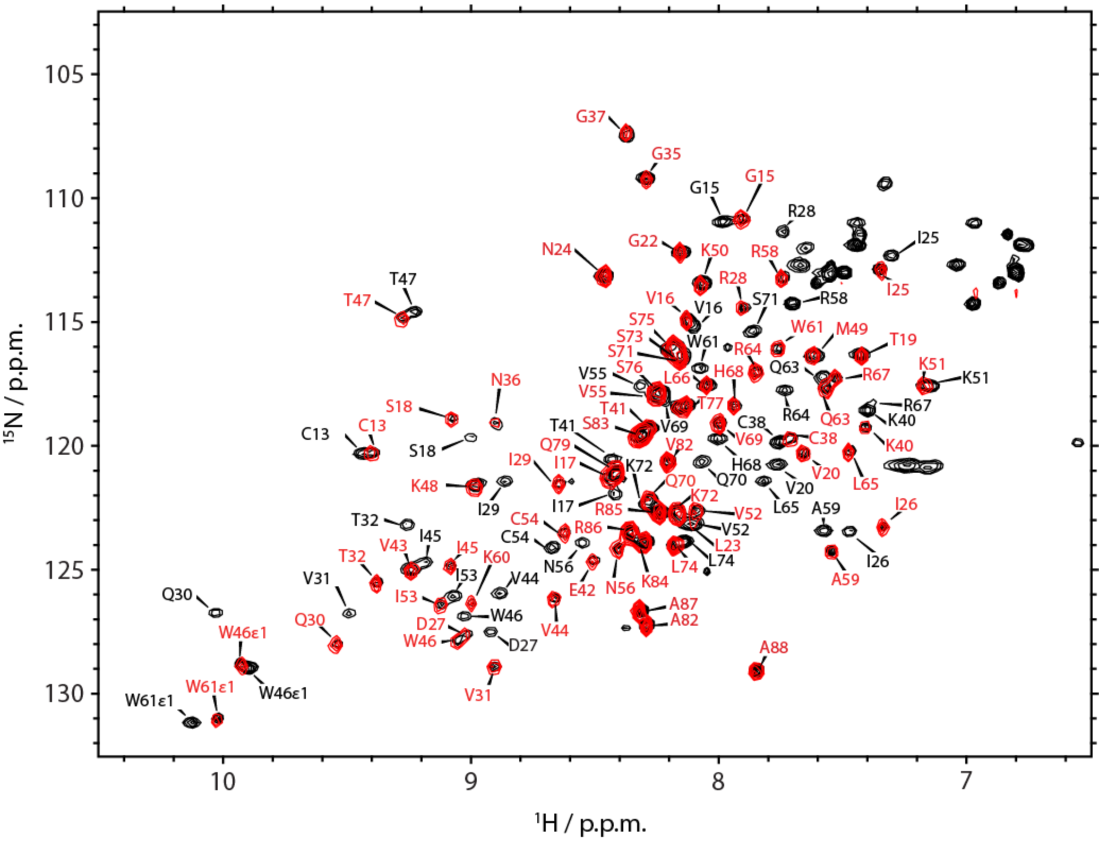

ΔN-CXCL13 dimerization in slow exchange regime. The 2D [^{15}N,^{1}H]-correlation spectra of ΔN-CXCL13 at 56 (red) and 860 (black) µM are overlaid. The resonances are assigned for the monomer and dimer forms of CXCL13. The specific resonances of the monomer are those present in both spectra. The specific resonances of the dimer form are only present in the spectrum of CXCL13 at 860 µM (black). The peaks displayed in blue are those used for K_D calculation.

Figure S8

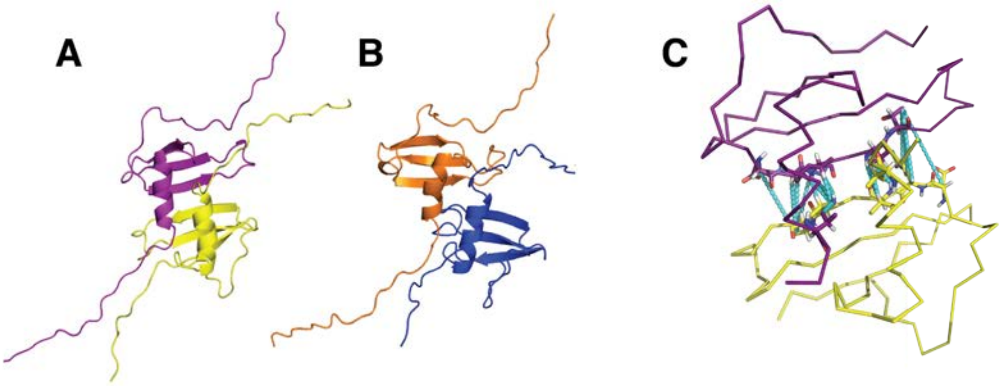

Molecular model of CXCL13 homodimer. **A, B**: CXCL13 homodimer was calculated by protein-protein docking using HADDOCK [14], in the manner of earlier modelling of dimers [15, 16]. This led to 11 representative models, of which two were identified as the best in initial evaluation possessing similar HADDOCK and z-scores, and overall backbone RMSD of 7 Å. Both monomeric chains are coloured differently. **C**: Intermolecular NOEs were extracted from 3D NOESY data recorded on sample containing both monomer and dimer forms of CXCL13 and used to further define critical protomer-protomer distances. Of the two dimer HADDOCK-derived models, model A (depicted as ribbon in same orientation as in **A**) satisfies intermolecular NOEs (dashed lines in *cyan*) extracted from 3D ^{15}N- and ^{13}C-edited NOESY data recorded on sample containing both monomer and dimer forms of CXCL13. This model was used in docking study with the top 25 ranked sequences of dp4 bound to CXCL13 monomer at binding site I (α-helix).

Figure S9

A

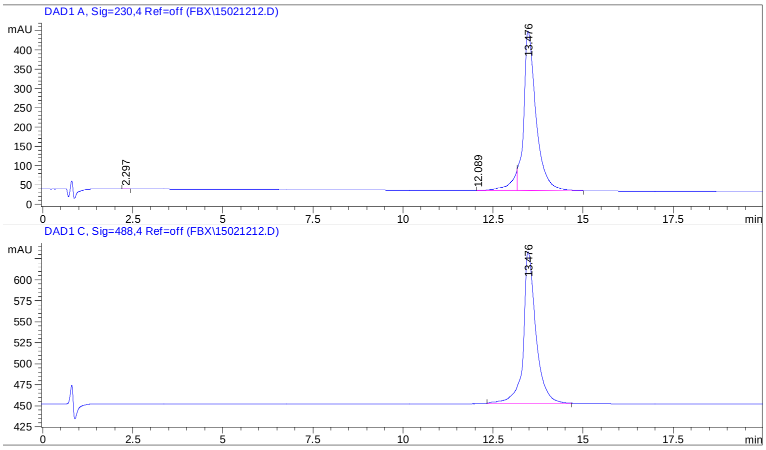

B

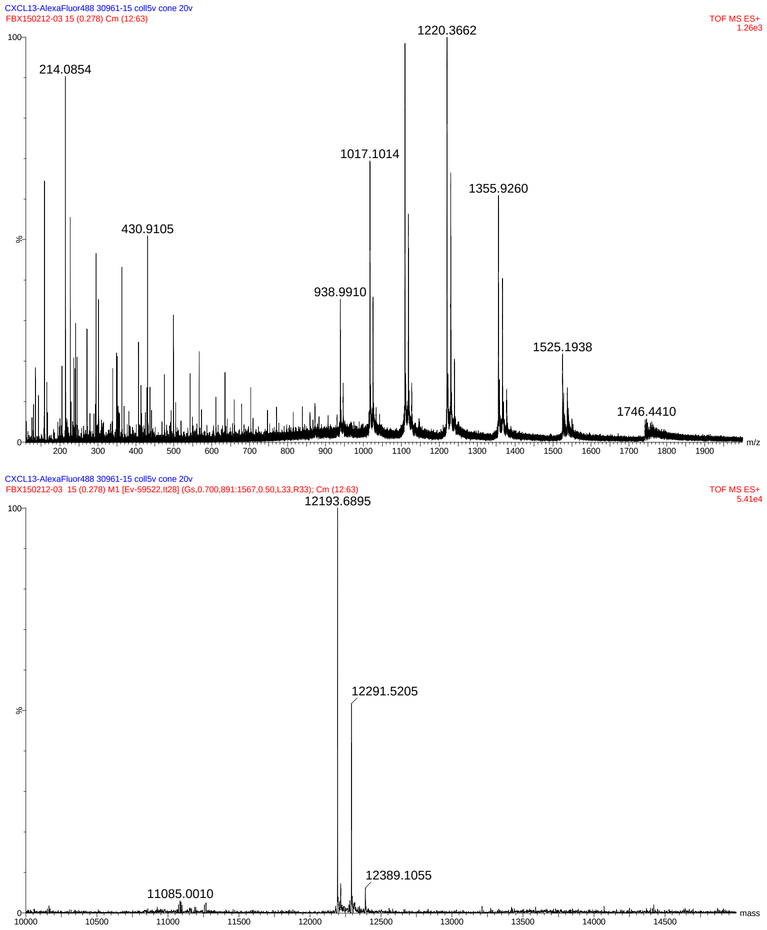

S6-tagged CXCL13 analysis. **A**: HPLC trace for Alexa Fluor 488 S6-tagged CXCL13. **B**: HR-Mass (upper graph: m/z fullscan lower graph: deconvolutated spectrum), performed using the positive mode indicated a molecular mass of 12193.6895 (expected average mass for $C_{536}H_{873}N_{154}O_{152}S_8P_1$: 12194.2761).

Figure S10

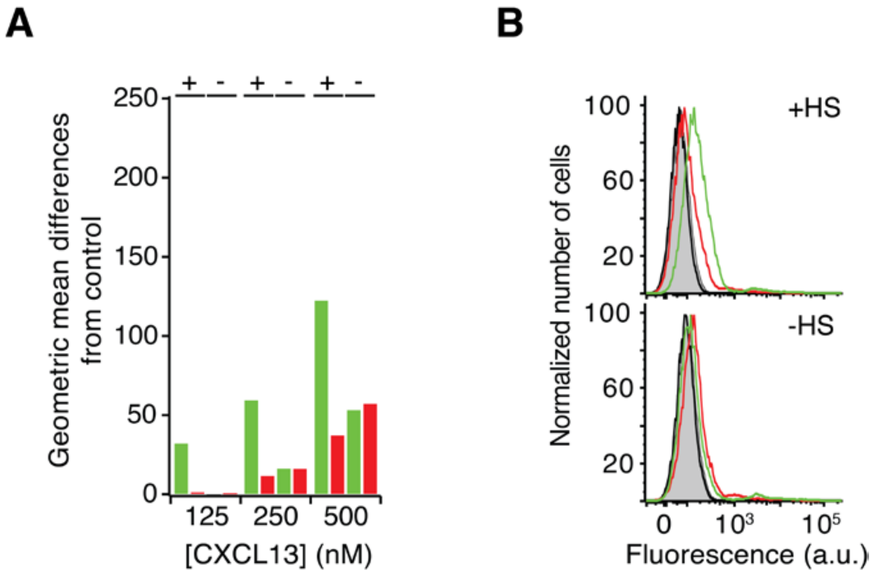

CXCL13 binding to HS$^+$ and HS$^-$ cells. The binding of fluorochrome-coupled CXCL13 over cell surfaces, featuring either HS or not, was assayed by flow cytometry. **A**: Parental CHO-K1 (+) or HS-deficient CHO-pgs-D677 cells (-) were incubated with either the wild type (green bars) or the double mutant (red bars) CXCL13 and the remained fluorescence after washing was then quantified. The deviations of fluorescence geometric mean from control (auto-fluorescence) were reported against the indicated concentration of CXCL13. **B**: Representative FACS histograms recorded on parental CHO-K1 (upper) or HS-deficient CHO-pgs-D677 (lower) cells following incubation with 500 nM of wild type (green) or double mutant (red) CXCL13 proteins.

Figure S11

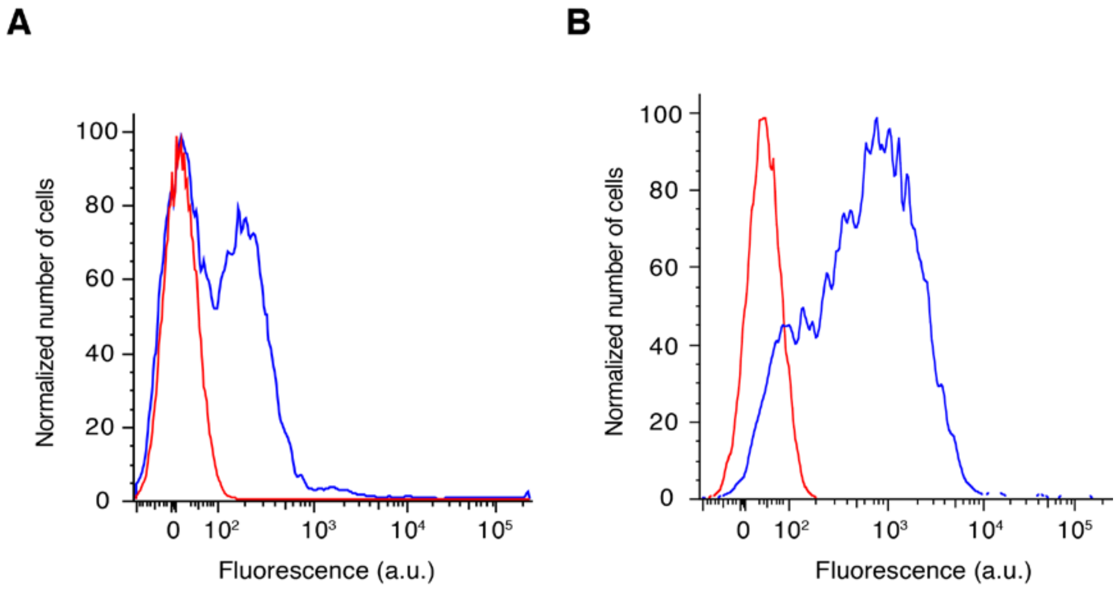

CXCR5 expression of splenocytes and transfected CHO cells. **A**: The levels of CXCR5, the CXCL13 receptor, expressed at the cellular surface of extracted mouse splenocytes and **B**: CXCR5-transfected CHO 677 cells, were assayed by FACS, based on the detection of cellular fluorescence subsequent to incubation with fluorochrome-conjugated antibody against CXCR5 (in blue), compared to auto-fluorescence (in red).

Figure S12

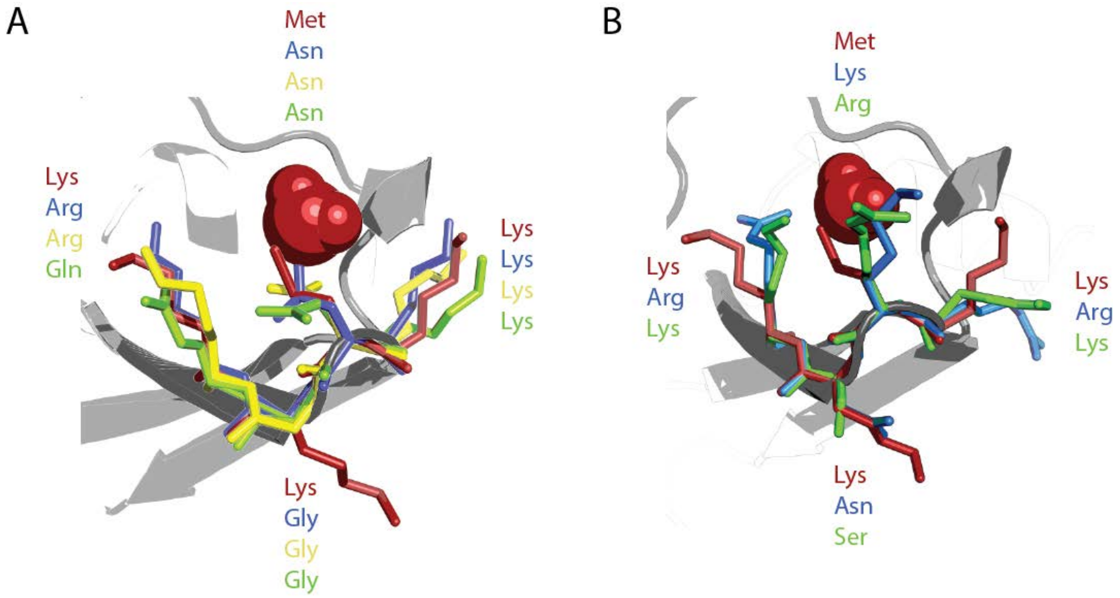

Chemokine's 40s loop comparison. Analysis of the 40s loop structure for both CXC- and CC- chemokines (CXCL1, CXCL2, CXCL4, CCL4 and CCL5) for which HS binding has been shown to involve this particular site [17-21]. **A**: For CXC-chemokines, both Lys48 and Lys51 of CXCL13 (in red) aligned very well with Lys45 and Arg48 of CXCL1 (in blue, pdb 1MGS), Lys45 of CXCL2 (in green, pdb 1QNK) and Lys46 and Arg49 of CXCL4 (in yellow, pdb 1RHP). **B**: For CC-chemokines, the Lys48 and Lys51 of CXCL13 aligned with Lys45 and Lys48 of CCL4 (in green, pdb 2X6L), and Lys44 and Arg47 of CCL5 (in blue, pdb 5COY). Alignments were achieved for backbone atoms only (rmsd < 0.8). The main notable difference is the Met49 of CXCL13 (which [CH3]ε group of CXCL13 is displayed as a red sphere) that aligned with an asparagine in the three CXC chemokines, or a basic residue in the two CC chemokine, localized in between the two other basic residues of this cluster. The methionine side chain conformation is defined according to the very intense NOE observed between Met-[CH3]ε and Ile25-[CH3]γ2. The presence of this bulky residue, localized within the basic sequence of the CXCL13 loop 40S is thus likely to hinder access to HS.

REFERENCES

1 Liu, H., Naismith, J. H. 2008 An efficient one-step site-directed deletion, insertion, single and multiple-site plasmid mutagenesis protocol. *BMC biotechnology*. **8**, 91. (10.1186/1472-6750-8-91)

2 Delaglio, F., Grzesiek, S., Vuister, G. W., Zhu, G., Pfeifer, J., Bax, A. 1995 NMRPipe: a multidimensional spectral processing system based on UNIX pipes. *Journal of biomolecular NMR*. **6**, 277-293.

3 Lee, W., Tonelli, M., Markley, J. L. 2015 NMRFAM-SPARKY: enhanced software for biomolecular NMR spectroscopy. *Bioinformatics (Oxford, England)*. **31**, 1325-1327. (10.1093/bioinformatics/btu830)

4 Senn, H., Werner, B., Messerle, B. A., Weber, C., Traber, R., Wiithrich, K. 1989 Stereospecific assignment of the methyl lH NMR lines of valine and leucine in polypeptides by nonrandom 13C labelling. *FEBS LETTERS*. **249**, 113-118.

5 Williamson, M. P. 2013 Using chemical shift perturbation to characterise ligand binding. *Prog Nucl Magn Reson Spectrosc*. **73**, 1-16. (10.1016/j.pnmrs.2013.02.001)

6 Farrow, N. A., Zhang, O., Forman-Kay, J. D., Kay, L. E. 1994 A heteronuclear correlation experiment for simultaneous determination of 15N longitudinal decay and chemical exchange rates of systems in slow equilibrium. *Journal of biomolecular NMR*. **4**, 727-734.

7 Rossi, P., Shi, L., Liu, G., Barbieri, C. M., Lee, H. W., Grant, T. D., Luft, J. R., Xiao, R., Acton, T. B., Snell, E. H., *et al.* 2015 A hybrid NMR/SAXS-based approach for discriminating oligomeric protein interfaces using Rosetta. *Proteins*. **83**, 309-317. (10.1002/prot.24719)

8 Kay, L. E., Torchia, D. A., Bax, A. 1989 Backbone dynamics of proteins as studied by 15N inverse detected heteronuclear NMR spectroscopy: application to staphylococcal nuclease. *Biochemistry*. **28**, 8972-8979.

9 D.A. Case, V. Babin, J.T. Berryman, R.M. Betz, Q. Cai, D.S. Cerutti, T.E. Cheatham, I., T.A. Darden, R.E. Duke, H. Gohlke, *et al.* 2014 AMBER 14. *University of California, San Francisco.*,

10 Miller, B. R., 3rd, McGee, T. D., Jr., Swails, J. M., Homeyer, N., Gohlke, H., Roitberg, A. E. 2012 MMPBSA.py: An Efficient Program for End-State Free Energy Calculations. *Journal of chemical theory and computation*. **8**, 3314-3321. (10.1021/ct300418h)

11 Case, D. A., Darden, T. A., Cheatham, I., T.E., Simmerling, C. L., Wang, J., Duke, R. E., Luo, R., Walker, R. C., Zhang, W., Merz, K. M., *et al.* 2012 AMBER 13. *University of California, San Francisco*.

12 Kawamura, T., Stephens, B., Qin, L., Yin, X., Dores, M. R., Smith, T. H., Grimsey, N., Abagyan, R., Trejo, J., Kufareva, I., *et al.* 2014 A general method for site specific fluorescent labeling of recombinant chemokines. *PloS one*. **9**, e81454. (10.1371/journal.pone.0081454)

13 Sarrazin, S., Bonnaffe, D., Lubineau, A., Lortat-Jacob, H. 2005 Heparan sulfate mimicry: a synthetic glycoconjugate that recognizes the heparin binding domain of interferon-gamma inhibits the cytokine activity. *The Journal of biological chemistry*. **280**, 37558-37564. (10.1074/jbc.M507729200)

14 van Zundert, G. C., Rodrigues, J. P., Trellet, M., Schmitz, C., Kastritis, P. L., Karaca, E., Melquiond, A. S., van Dijk, M., de Vries, S. J., Bonvin, A. M. 2016 The HADDOCK2.2 Web Server: User-Friendly Integrative Modeling of Biomolecular Complexes. *Journal of molecular biology*. **428**, 720-725. (10.1016/j.jmb.2015.09.014)

15 Kaczor, A. A., Selent, J., Sanz, F., Pastor, M. 2013 Modeling Complexes of Transmembrane Proteins: Systematic Analysis of ProteinProtein Docking Tools. *Molecular informatics*. **32**, 717-733. (10.1002/minf.201200150)

16 Sudhakar, D. R., P, K., Subbarao, N. 2016 Docking and molecular dynamics simulation study of EGFR1 with EGF-like peptides to understand molecular interactions. *Molecular bioSystems*. **12**, 1987-1995. (10.1039/c6mb00032k)

17 Fairbrother, W. J., Reilly, D., Colby, T. J., Hesselgesser, J., Horuk, R. 1994 The solution structure of melanoma growth stimulating activity. *Journal of molecular biology*. **242**, 252-270. (10.1006/jmbi.1994.1577)

18 Liang, W. G., Triandafillou, C. G., Huang, T. Y., Zulueta, M. M., Banerjee, S., Dinner, A. R., Hung, S. C., Tang, W. J. 2016 Structural basis for oligomerization and glycosaminoglycan binding of CCL5 and CCL3. *Proceedings of the National Academy of Sciences of the United States of America*. **113**, 5000-5005. (10.1073/pnas.1523981113)

19 Qian, Y. Q., Johanson, K. O., McDevitt, P. 1999 Nuclear magnetic resonance solution structure of truncated human GRObeta [5-73] and its structural comparison with CXC chemokine family members GROalpha and IL-8. *Journal of molecular biology*. **294**, 1065-1072. (10.1006/jmbi.1999.3333)

20 Ren, M., Guo, Q., Guo, L., Lenz, M., Qian, F., Koenen, R. R., Xu, H., Schilling, A. B., Weber, C., Ye, R. D., *et al.* 2010 Polymerization of MIP-1 chemokine (CCL3 and CCL4) and clearance of MIP-1 by insulin-degrading enzyme. *The EMBO journal*. **29**, 3952-3966. (10.1038/emboj.2010.256)
21 Zhang, X., Chen, L., Bancroft, D. P., Lai, C. K., Maione, T. E. 1994 Crystal structure of recombinant human platelet factor 4. *Biochemistry*. **33**, 8361-8366.
